# Design, Synthesis, and Antiprotozoal Evaluation of New Promising 2,9-*Bis*[(substituted-aminomethyl)]-4,7-phenyl-1,10-phenanthroline Derivatives, a Potential Alternative Scaffold to Drug Efflux

**DOI:** 10.3390/pathogens11111339

**Published:** 2022-11-13

**Authors:** Jean Guillon, Anita Cohen, Clotilde Boudot, Sarah Monic, Solène Savrimoutou, Stéphane Moreau, Sandra Albenque-Rubio, Camille Lafon-Schmaltz, Alexandra Dassonville-Klimpt, Jean-Louis Mergny, Luisa Ronga, Mikel Bernabeu de Maria, Jeremy Lamarche, Cristina Dal Lago, Eric Largy, Valérie Gabelica, Serge Moukha, Pascale Dozolme, Patrice Agnamey, Nadine Azas, Catherine Mullié, Bertrand Courtioux, Pascal Sonnet

**Affiliations:** 1 Faculty of Pharmacy, University of Bordeaux, CNRS, INSERM, ARNA, UMR 5320, U1212, F-33000 Bordeaux, France; 2 Faculty of Pharmacy, University of Aix-Marseille, IRD, AP-HM, SSA, VITROME, F-13005 Marseille, France; 3 Faculty of Pharmacy, Institute of Neuroepidemiology and Tropical Neurology, University of Limoges, INSERM U1094, F-87025 Limoges, France; 4 Faculty of Pharmacy, Agents Infectieux, Résistance et Chimiothérapie (AGIR), UR 4294, UFR de Pharmacie, University of Picardie Jules Verne, F-80037Amiens, France; 5 Laboratoire d’Optique et Biosciences, Institut Polytechnique de Paris, Ecole Polytechnique, CNRS, INSERM, F- 91128 Palaiseau, France; 6 Université de Pau et des Pays de l’Adour, E2S UPPA, CNRS, IPREM, F-64012 Pau, France; 7 University of Bordeaux, CNRS, INSERM, ARNA, UMR 5320, U1212, IECB, F-33600 Pessac, France; 8 Centre de Recherche Cardio-thoracique de Bordeaux (CRCTB), UMR U1045 INSERM, PTIB-Hôpital Xavier Arnozan, F-33600 Pessac, France; 9 INRAE Bordeaux Aquitaine, F- 33140 Villenave-d’Ornon, France

**Keywords:** antimalarial activity, phenanthroline, G-quadruplex, antileishmanial activity, antitrypanosomal activity

## Abstract

A series of novel 2,9-*bis*[(substituted-aminomethyl)]-4,7-phenyl-1,10-phenanthroline derivatives was designed, synthesized, and evaluated in vitro against three protozoan parasites (*Plasmodium falciparum*, *Leishmania donovani* and *Trypanosoma brucei brucei*). Pharmacological results showed antiprotozoal activity with IC_50_ values in the sub and μM range. In addition, the in vitro cytotoxicity of these original molecules was assessed with human HepG2 cells. The substituted diphenylphenanthroline **1l** was identified as the most potent antimalarial derivative with a ratio of cytotoxic to antiparasitic activities of 505.7 against the *P. falciparum* CQ-resistant strain W2. Against the promastigote forms of *L. donovani*, the phenanthrolines **1h**, **1j**, **1n** and **1o** were the most active with IC_50_ from 2.52 to 4.50 μM. The phenanthroline derivative **1o** was also identified as the most potent trypanosomal candidate with a selectivity index (SI) of 91 on *T. brucei brucei* strain. FRET melting and native mass spectrometry experiments evidenced that the nitrogen heterocyclic derivatives bind the telomeric G-quadruplexes of *P. falciparum* and *Trypanosoma*. Moreover, as the telomeres of the parasites *P. falciparum* and *Trypanosoma* could be considered to be possible targets of this kind of nitrogen heterocyclic derivatives, their potential ability to stabilize the parasitic telomeric G-quadruplexes have been determined through the FRET melting assay and by native mass spectrometry.

## 1. Introduction

At present, no single tool is available to solve the public health problem of malaria [1], which caused 241 million cases and 627,000 deaths in 2020 worldwide [2]. This mortality indicator is significantly lower than in 2000 (896,000 deaths) even if there were an estimated 14 million more malaria cases and 47,000 more deaths in 2020 compared to 2019 due to disruptions to services during the pandemic [2]. In response, the World Health Organization (WHO) recently updated its recommendations, particularly in terms of prevention, which includes preventive chemotherapies, vector control and vaccination [3]. (i) Indeed, young children still represent a significant proportion of the population at risk as the percentage of total malaria deaths in children aged under 5 years reduced from 87% in 2000 to 77% in 2020 [1]. To contribute to their prevention, since October 2021, the WHO recommended that the RTS,S/AS01 malaria vaccine be used for the prevention of *P. falciparum* malaria in children living in regions with moderate to high transmission [4]. If introduced widely and urgently, the RTS,S/AS01 vaccine could save thousands of children’s lives every year. (ii) Moreover, concerning the vector control, manufacturers delivered about 229 million insecticide-treated mosquito nets to malaria endemic countries in 2020 [2]. Of these, an increasing percentage of 19.4% was represented by pyrethroid–piperonyl butoxide nets (12.4% more than in 2019) and 5.2% were dual active ingredient insecticide-treated mosquito nets (3.6% more than in 2019) [2]. Then, the percentage of the population sleeping under an insecticide-treated mosquito net increased considerably between 2000 and 2020, for the whole population (from 2% to 43%) and for the population at risk (from 3% to 49% for children aged under 5 years and from 3% to 49% for pregnant women) [2]. (iii) Chemoprevention strategies are also currently recommended and evaluated by the WHO [5].

Nevertheless, despite these numerous prevention efforts, the increasing multidrug resistance of *Plasmodium* parasites worldwide is worrying and contributes strongly to the WHO’s concern that the 2030 targets of the WHO’s global malaria strategy will not be met [2]. Indeed, molecular markers of *Plasmodium falciparum* resistance to antimalarial drugs have been found to be associated with reduced treatment response and are useful in surveillance for resistance [5]. Thus, among the tools to be developed and prioritized to combat malaria, more effective antimalarial medicines with new potential mechanisms of action are urgently needed [6].

**Figure 1:**
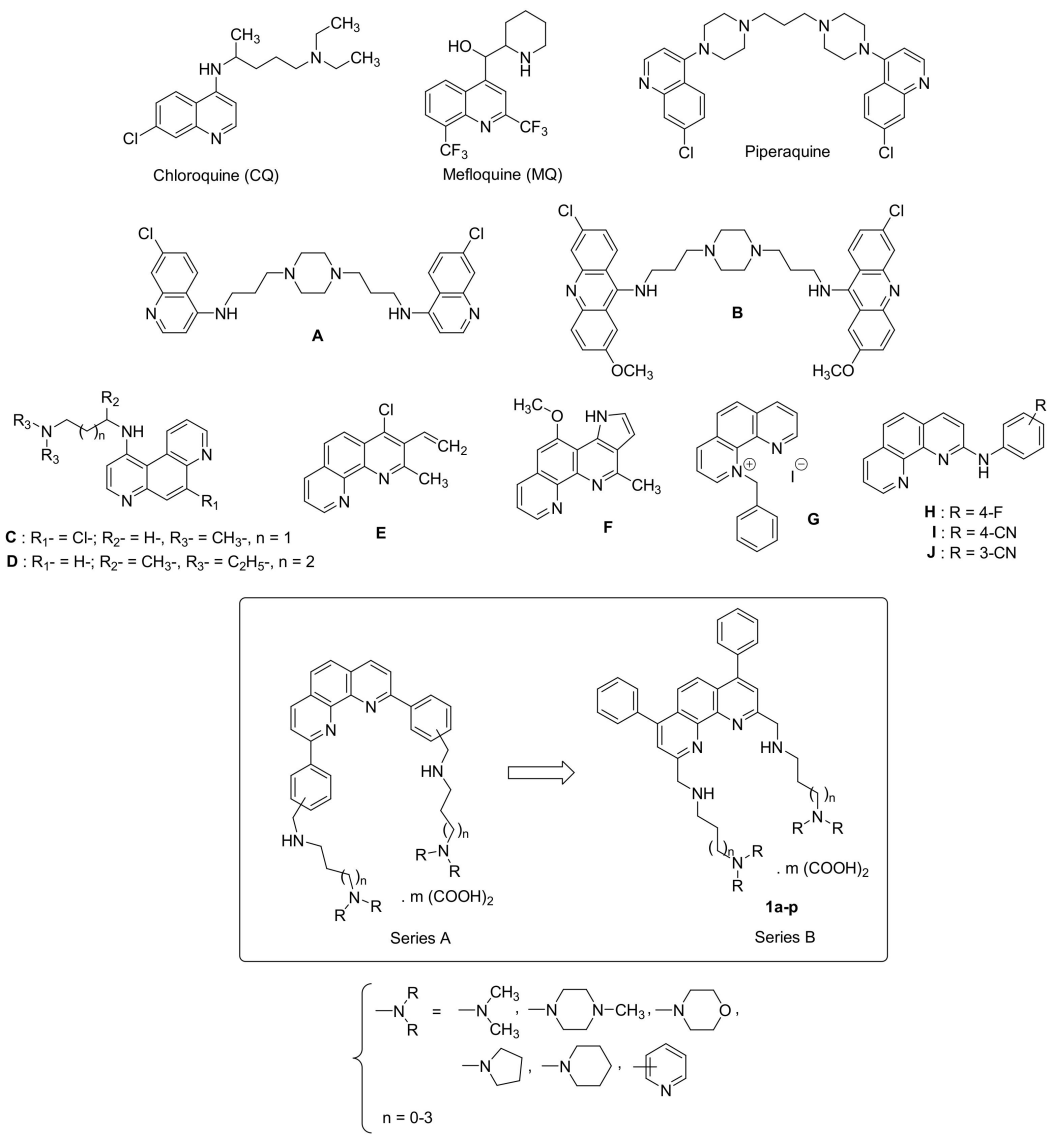
The structures of chloroquine (CQ), mefloquine (MQ), piperaquine, bisquinoline **A**, bisacridine **B**, 4,7-phenanthrolines **C** and **D**, 4-chloro-2-methyl-3-vinyl-1,10-phenanthroline **E**, 4-methoxy-11-methyl-3*H*-pyrrolo [3,2-*c*] [1,10]phenanthroline **F**, *N*-benzyll-1,10-phenanthroline **G**, anilinophenanthrolines **H**–**J** and newly synthesized substituted phenanthroline derivatives **1**.

In this context, efforts to discover new 4-aminoquinoline derivatives are ongoing. Indeed, it is unlikely that the parasite will be able to evolve resistance to drugs targeting the pathway involved in hemoglobin degradation. Previous studies showed that modulation and modification of the lateral side chain of chloroquine (CQ) or of mefloquine (MQ) that led to original aminoquinoline compounds avoid the CQ resistance mechanism [7,8,9,10]. Another original strategy is to design and synthesize quinoline-based drugs that are not recognized by the protein system involved in the drug efflux. The efflux pumps serve both as natural defense mechanisms and influence the bioavailability and disposition of drugs. Initially, such mechanism was suggested in *Plasmodium falciparum* where erythrocytes infected with CQ-resistant parasites accumulated significantly less drug than the sensitive ones [11], before its further study led to the identification of the *Pfcrt* gene [12] among other quinoline drug resistance mechanisms [13]. Such new series not recognized by the protein system involved in the drug efflux of bisquinoline and bisacridine antimalarial drugs (Figure 1A,B and Piperaquine) have been described in the literature [9,14,15,16]. These new derivatives had much lower resistance indices than CQ, indicating that these *bis*-heterocyclic structures are less efficiently rejected by efflux by drug-resistant parasites. Moreover, previous studies on in vitro antiplasmodial activity of diaza phenanthrene analogs indicated that the 1,10-phenanthroline skeleton represents an interesting and potential antimalarial lead compound. A few bioactive antimalarial phenanthrolines based on this skeleton have been described such as 4,7-phenanthrolines Figure 1C,D [17], 4-chloro-2-methyl-3-vinyl-1,10-phenanthroline Figure 1E [18], 4-methoxy-11-methyl-3*H*-pyrrolo [3,2-*c*] [1,10]phenanthroline Figure 1F [19], and *N*-benzyl-1,10-phenanthrolinium iodide **G** (Figure 1) [20,21]. Moreover, the study of the trypanocidal activity of new anilinophenanthrolines Figure 1H,J against *Trypanosoma cruzi* has also been reported [22].

Nevertheless, among the protozoa of medical interest, trypanosomatid parasites are also important, and responsible for tropical diseases, mainly leishmaniases caused by more than 20 *Leishmania* species described to be infective to humans, and trypanosomiases including human African trypanosomiasis (or sleeping sickness) caused by *Trypanosoma brucei* and South American trypanosomiasis (or Chagas disease) caused by *Trypanosoma cruzi*. Classified as neglected tropical diseases according to the WHO, which wants to eliminate them as a public health problem by 2030 [23], their impact is even more important because of the current limited therapeutic arsenal which is highly toxic and subject to the development of increasingly drug-resistant parasite strains [24].

During our work, which is focused on the discovery of new nitrogen heterocyclic derivatives that can be used in antiprotozoal chemotherapy [25,26,27,28,29,30], we previously designed and synthesized a series of substituted 2,9-*bis*[(substituted-aminomethyl)phenyl]-1,10-phenanthroline derivatives (series A; Figure 1) designed as antimalarial candidates, which could bind to *Plasmodium falciparum* DNA G-quadruplexes [31] in order to bypass the resistance mechanisms developed by the parasites and based on efflux. By considering our experience in the field of the synthesis of new antiprotozoal heterocyclic compounds, we describe here the design and synthesis of new 2,9-*bis*[(substituted-aminomethyl)]-4,7-phenyl-1,10-phenanthroline derivatives salts **1** (Series B; Figure 1) that could be considered to be new bio-isoster analogs of our previously described phenanthrolines series A. We report on their in vitro antiplasmodial activity against the CQ-sensitive (3D7) and the CQ-resistant (W2) strains of the malaria parasite *P. falciparum*. As we could consider that nitrogen heterocyclic pharmacophores are the fundamental moieties of several antiprotozoan candidates, these quinoline-like analogues were also tested for in vitro efficacy against medically important protozoans *Leishmania donovani* and *Trypanosoma brucei brucei*.

In addition, the in vitro cytotoxicity of our new 2,9-*bis*[(substituted-aminomethyl)]-4,7-phenyl-1,10-phenanthroline derivatives **1** was assessed in human HepG2 cells, and a selectivity index, the ratio of cytotoxic to antiparasitic activity, was determined for each derivative. The telomeres of the different protozoa could constitute attractive drug targets [32,33,34,35] in order to bypass the resistance mechanisms based on efflux and telomerase activity is detected in gametocytes and during the transition to the erythrocytic stage of *P. falciparum* [36]. The telomeric 3′ G-overhang region of *P. falciparum* is a repetition of degenerate unit 5′GGGTTYA3′ (where Y could be T or C) [37] which can fold into intramolecular G-quadruplex [38]. This difference between parasitic and human (5′GGGTTA3′) G-quadruplexes is also observed with *L.* spp and *T. brucei brucei*, which augurs the possibility of developing antiparasitic ligands targeting specifically G-quadruplexes found in these protozoal species. Thus, we investigated whether these derivatives could stabilize some parasitic telomeric DNA G-quadruplex structures. Consequently, potential stabilization of *P. falciparum* and *T. brucei brucei* telomeric G-quadruplexes was evaluated using a FRET melting assay and by native mass spectrometry.

## 2. Materials and Methods

### 2.1. Chemistry

#### 2.1.1. General

Commercially available reagents were used as received without additional purification. Melting points were determined with an SM-LUX-POL Leitz hot-stage microscope (Leitz GMBH, Midland, ON, USA) and are uncorrected. NMR spectra were recorded with tetramethylsilane as an internal standard using a BRUKER AVANCE 300 spectrometer (Bruker BioSpin, Wissembourg, France). Splitting patterns have been reported as follows: s = singlet; bs = broad singlet; d = doublet; t = triplet; q = quartet; dd = double doublet; ddd = double double doublet; qt = quintuplet; m = multiplet. Analytical TLC were carried out on 0.25 precoated silica gel plates (POLYGRAM SIL G/UV254) and visualization of compounds after UV light irradiation. Silica gel 60 (70–230 mesh) was used for column chromatography. Mass spectra were recorded on an ESI LTQ Orbitrap Velos mass spectrometer (ThermoFisher, Bremen, Germany). Ionization was performed using an Electrospray ion source operating in positive ion mode with a capillary voltage of 3.80 kV and capillary temperature of 250 °C. The scan type analyzed was full scan, all MS recordings were in the m/z range between 150 and 2000 m/z. None type of fragmentation was carried out and the resolution used for the analysis was 60.000.

#### 2.1.2. Synthesis of the 2,9-bis(formyl)-4,7-diphenyl-1,10-phenanthroline (2)

A mixture of bathocuproine (1.0 g, 2.8 mmol) and SeO_2_ (0.75 g, 6.76 mmol) in 17 mL of dioxane was heated at 80 °C for 2 h. The reaction mixture was then filtered, washed with hot dioxane and the filtrate evaporated to dryness. The residue was triturated in ethanol, filtered, and purified by column chromatography on silica gel using dichloromethane/methanol (90/10: *v*/*v*) as eluent to give **2**. Beige cristals (83%); Mp = 264 °C 1H NMR (CDCl3) δ: 10.64 (s, 2H, 2 CHO), 8.35 (s, 2H, H-3 and H-8), 8.12 (s, 2H, H-5 and H-6), 7.60–7.58 (m, 10H, H-phenyl).

#### 2.1.3. General Procedure for the Synthesis of 2,9-Bis[(substituted-iminomethyl)phenyl]-4,7-diphenyl-1,10-phenanthrolines (**3a-p**)

The 2,9-bis(formyl)-4,7-diphenyl-1,10-phenanthroline **2** (150 mg, 0.386 mmol) was dissolved in 6 mL of toluene. Activated molecular sieves 4 Å (800 mg) were introduced and then dialkylamine (0.812 mmol). The reaction mixture was stirred in a stoppered flask 24 h. The suspension that was obtained was filtered, washed with dichloromethane, then the solvent was removed under reduced pressure to afford the diimine **3**. Products were then used without further purification.

##### 2,9-*Bis*[(2-dimethylaminoethyl)iminomethyl]-4,7-diphenyl-1,10-phenanthroline (**3a**)

Orange oil, (71%). 1H NMR (CDCl_3_) δ: 8.99 (s, 2H, HC=N), 8.42 (s, 2H, H-3 and H-8), 7.94 (s, 2H, H-5 and H-6), 7.57–7.52 (m, 10H, H-phenyl), 3.94 (t, 4H, *J* = 6.90 Hz, 2 NCH_2_), 2.73 (t, 4H, *J* = 6.90 Hz, 2 NCH_2_), 2.36 (s, 12H, 2 N(CH_3_)_2_).

##### 2,9-*Bis[*(3-dimethylaminopropyl)iminomethyl]-4,7-diphenyl-1,10-phenanthroline (**3b**)

Orange oil (98%). 1H NMR (CDCl_3_) δ: 8.93 (s, 2H, 2 HC=N), 8.38 (s, 2H, H-3 and H-8), 7.91 (s, 2H, H-5 and H-6), 7.57–7.42 (m, 10H, H-phenyl), 3.82 (t, 4H, *J* = 6.90 Hz, 2 NCH_2_), 2.40 (t, 4H, *J* = 6.90 Hz, 2 NCH_2_), 2.24 (s, 12H, 2 N(CH_3_)_2_), 1.94 (qt, 4H, *J* = 6.90 Hz, 2 CH_2_).

##### 2,9-*Bis*[(4-dimethylaminobutyl)iminomethyl]-4,7-diphenyl-1,10-phenanthroline (**3c**)

Orange oil (98%). 1H NMR (CDCl_3_) δ: 8.93 (s, 2H, 2 HC=N), 8.37 (s, 2H, H-3 and H-8), 7.91 (s, 2H, H-5 and H-6), 7.57–7.47 (m, 10H, H-phenyl), 3.80 (t, 4H, *J* = 6.90 Hz, 2 NCH_2_), 2.34 (t, 4H, *J* = 6.90 Hz, 2 NCH_2_), 2.21 (s, 12H, 2 N(CH_3_)_2_), 1.82–1.73 (m, 4H, 2 CH_2_), 1.63–1.53 (m, 4H, 2 CH_2_).

##### 2,9-*Bis*[(5-dimethylaminopentyl)iminomethyl]-4,7-diphenyl-1,10-phenanthroline (**3d**)

Yellow oil (97%). 1H NMR (CDCl_3_) δ: 8.94 (s, 2H, 2 HC=N), 8.40 (s, 2H, H-3 and H-8), 7.94 (s, 2H, H-5 and H-6), 7.59–7.53 (m, 10H, H-phenyl), 3.81 (t, 4H, *J* = 6.90 Hz, 2 NCH_2_), 2.27 (t, 4H, *J* = 6.90 Hz, 2 NCH_2_), 2.23 (s, 12H, 2 N(CH_3_)_2_), 1.81 (qt, 4H, *J* = 6.90 Hz, 2 CH_2_), 1.56–1.53 (m, 4H, 2 CH_2_), 1.48–1.45 (m, 4H, 2 CH_2_).

##### 2,9-*Bis* [2-(4-methylpiperazin-1-yl)ethyl)iminomethyl]-4,7-diphenyl-1,10-phenanthroline (**3e**)

Orange oil, (98%). 1H NMR (CDCl_3_) δ: 8.97 (s, 2H, 2 HC=N), 8.36 (s, 2H, H-3 and H-8), 7.93 (s, 2H, H-5 and H-6), 7.57–7.50 (m, 10H, H-phenyl), 3.97 (t, 4H, *J* = 6.90 Hz, 2 NCH_2_), 2.80 (t, 4H, *J* = 6.90 Hz, 2 NCH_2_), 2.65–2.45 (m, 16H, 8 NCH_2_pip), 2.35 (s, 6H, 2 NCH_3_).

##### 2,9-*Bis*[[(3-(4-methylpiperazin-1-yl)propyl)iminomethyl]-4,7-diphenyl-1,10-phenanthroline (**3f**)

Orange oil (98%). 1H NMR (CDCl_3_) δ: 8.88 (s, 2H, 2 HC=N), 8.33 (s, 2H, H-3 and H-8), 7.87 (s, 2H, H-5 and H-6), 7.50–7.45 (m, 10H, H-phenyl), 3.77 (t, 4H, *J* = 6.90 Hz, 2 NCH_2_), 2.46–2.32 (m, 20H, 10 NCH_2_), 2.21 (s, 6H, 2 NCH_3_), 1.95–1.91 (qt, 4H, *J* = 6.90 Hz, 2 CH_2_).

##### 2,9-*Bis*[[(4-(4-methylpiperazin-1-yl)butyl)iminomethyl]-4,7-diphenyl-1,10-phenanthroline (**3g**)

Orange oil (98%). 1H NMR (CDCl_3_) δ: 8.89 (s, 2H, 2 HC=N), 8.33 (s, 2H, H-3 and H-8), 7.87 (s, 2H, H-5 and H-6), 7.53–7.41 (m, 10H, H-phenyl), 3.77 (t, 4H, *J* = 6.90 Hz, 2 NCH_2_), 2.42–2.26 (m, 20H, 10 NCH_2_pip), 2.22 (s, 6H, 2 NCH_3_), 1.80–1.70 (m, 4H, 2 CH_2_), 1.62–1.54 (m, 4H, 2 CH_2_).

##### 2,9-Bis [2-(morpholin-1-yl)ethyl)iminomethyl]-4,7-diphenyl-1,10-phenanthroline (**3h**)

Orange oil, (98%). 1H NMR (CDCl_3_) δ: 8.96 (s, 2H, 2 HC=N), 8.37 (s, 2H, H-3 and H-8), 7.93 (s, 2H, H-5 and H-6), 7.55–7.50 (m, 10H, H-phenyl), 3.96 (t, 4H, *J* = 6.90 Hz, 2 NCH_2_), 3.74 (t, 8H, *J* = 4.70 Hz, 4 OCH_2_), 2.79 (t, 4H, *J* = 6.90 Hz, 2 NCH_2_), 2.58 (t, 8H, *J* = 4.35 Hz,4 NCH_2_morph).

##### 2,9-Bis [3-(morpholin-1-yl)propyl)iminomethyl]-4,7-diphenyl-1,10-phenanthroline (**3i**)

Orange oil, (85%). 1H NMR (CDCl_3_) δ: 8.94 (s, 2H, 2 HC=N), 8.38 (s, 2H, H-3 and H-8), 7.93 (s, 2H, H-5 and H-6), 7.59–7.48 (m, 10H, H-phenyl), 3.84 (t, 4H, *J* = 6.90 Hz, 2 NCH_2_), 3.73 (t, 8H, *J* = 4.70 Hz, 4 OCH_2_), 2.51–2.45 (m, 12H, 2 NCH_2_ + 4 NCH_2_morph), 1.97 (qt, 4H, *J* = 6.90 Hz, 2 CH_2_).

##### 2,9-*Bis* [2-(piperidin-1-yl)ethyl)iminomethyl]-4,7-diphenyl-1,10-phenanthroline (**3j**)

Orange oil, (95%). 1H NMR (CDCl_3_) δ: 8.98 (s, 2H, 2 HC=N), 8.39 (s, 2H, H-3 and H-8), 7.93 (s, 2H, H-5 and H-6), 7.56–7.50 (m, 10H, H-phenyl), 3.98 (t, 4H, *J* = 6.90 Hz, 2 NCH_2_), 2.76 (t, 4H, *J* = 6.90 Hz, 2 NCH_2_), 2.56–2.52 (m, 8H, 4 NCH_2_pip), 1.65–1.61 (m, 8H, NCH_2_pip), 1.49–1.45 (m, 4H, 2 CH_2_pip).

##### 2,9-*Bis* [3-(piperidin-1-yl)propyl)iminomethyl]-4,7-diphenyl-1,10-phenanthroline (**3k**)

Yellow-orange oil, (98%). 1H NMR (CDCl3) δ: 8.94 (s, 2H, 2 HC=N), 8.37 (s, 2H, H-3 and H-8), 7.91 (s, 2H, H-5 and H-6), 7.57–7.48 (m, 10H, H-phenyl), 3.82 (t, 4H, *J* = 6.90 Hz, 2 NCH_2_), 2.43–2.37 (m, 12H, 2 NCH_2_ and 4 NCH_2_pip), 1.97 (qt, 4H, *J* = 6.90 Hz, 2 CH_2_), 1.62–1.56 (m, 8H, 4 CH_2_pip), 1.43–1.40 (m, 4H, 2 CH_2_pip).

##### 2,9-*Bis* [3-(pyrrolidin-1-yl)propyl)iminomethyl]-4,7-diphenyl-1,10-phenanthroline (**3l**)

Yellow-orange oil, (97%). 1H NMR (CDCl_3_) δ: 8.95 (s, 2H, 2 HC=N), 8.40 (s, 2H, H-3 and H-8), 7.94 (s, 2H, H-5 and H-6), 7.58–7.52 (m, 10H, H-phenyl), 3.86 (t, 4H, *J* = 6.90 Hz, 2 NCH_2_), 2.56 (t, 4H, *J* = 6.90 Hz, 2 NCH_2_pyrr), 2.55–2.52 (m, 8H, 4 NCH_2_pyrr), 2.01 (qt, 4H, *J* = 6.90 Hz, 2 CH_2_), 1.81–1.78 (m, 8H, 4 CH_2_pyrr).

##### 2,9-*Bis* [2-(pyridin-2-yl)ethyl)iminomethyl]-4,7-diphenyl-1,10-phenanthroline (**3m**)

Orange oil, (92%). 1H NMR (CDCl_3_) δ: 8.91 (s, 2H, 2 HC=N), 8.57 (m, 2H, 2 H-6pyr), 8.38 (s, 2H, H-3 and H-8), 7.91 (s, 2H, H-5 and H-6), 7.60–7.47 (m, 12H, H-phenyl, H-4pyr), 7.25–7.08 (m, 4H, H-3pyr, H-5pyr), 4.22 (t, 4H, *J* = 7.20 Hz, 2 NCH_2_), 3.28 (t, 4H, *J* = 7.20 Hz, 2 CH_2_pyr).

##### 2,9-Bis [2-(pyridin-3-yl)ethyl)iminomethyl]-4,7-diphenyl-1,10-phenanthroline (**3n**)

Orange oil, (93%). 1H NMR (CDCl_3_) δ: 8.84 (s, 2H, 2 HC=N), 8.53 (ddd, 2H, *J* = 1.90, 0.90 and 0.9 Hz, 2 H-2pyr), 8.44 (dd, 2H, *J* = 5.90 and 1.50 Hz,2 H-6pyr), 8.37 (s, 2H, H-3 and H-8), 7.93 (s, 2H, H-5 and H-6), 7.58–7.48 (m, 12H, 10 H-phenyl and 2 H-4pyr), 7.28–7.09 (m, 2H, H-5pyr), 4.04 (t, 4H, *J* = 6.60 Hz, 2 NCH_2_), 3.10 (t, 4H, *J* = 6.60 Hz, 2 CH_2_pyr).

##### 2,9-*Bis* [2-(pyridin-4-yl)ethyl)iminomethyl]-4,7-diphenyl-1,10-phenanthroline (**3o**)

Orange oil, (93%). 1H NMR (CDCl_3_) δ: 8.88 (s, 2H, 2 HC=N), 8.51 (dd, 4H, *J* = 6.00 and 1.50 Hz, H-2pyr and H-6pyr), 8.37 (s, 2H, H-3 and H-8), 7.95 (s, 2H, H-5 and H-6), 7.57–7.50 (m, 10H, H-phenyl), 7.25–7.13 (dd, 4H, *J* = 6.00 and 1.50 Hz, H-3pyr and H-5pyr), 4.07 (t, 4H, *J* = 6.90 Hz, 2 NCH_2_), 3.10 (t, 4H, *J* = 6.90 Hz, 2 CH_2_pyr).

##### 2,9-*Bis* [3-(pyridin-4-yl)propyl)iminomethyl]-4,7-diphenyl-1,10-phenanthroline (**3p**)

Yellow oil, (93%). 1H NMR (CDCl_3_) δ: 9.03 (s, 2H, 2 HC=N), 8.53 (dd, 4H, *J* = 6.00 and 1.50 Hz, H-2pyr and H-6pyr), 8.46 (s, 2H, H-3 and H-8), 7.97 (s, 2H, H-5 and H-6), 7.59–7.53 (m, 10H, H-phenyl), 7.15 (dd, 4H, *J* = 6.00 and 1.50 Hz, H-3pyr and H-5pyr), 3.88 (t, 4H, *J* = 7.20 Hz, 2 NCH_2_), 2.79 (t, 4H, *J* = 7.20 Hz, 2 CH_2_pyr), 2.16 (qt, 4H, *J* = 7.20 Hz, 2 CH_2_).

#### 2.1.4. General Procedure for the Synthesis of 2,9-Bis[(substituted-aminomethyl)phenyl]-4,7-diphenyl-1,10-phenanthrolines **4a-p**

To a solution of compound **3a**–**p** (0.4 mmol) in methanol (10 mL), sodium borohydride was added portion-wise at 0 °C (2.4 mmol, 6 eq.). The reaction mixture was then stirred at room temperature for 2 h. Then it was evaporated to dryness under reduced pressure. After cooling, the residue was triturated in water and extracted with dichloromethane (40 mL). The organic layer was separated, dried over sodium sulfate and activated charcoal, and evaporated to dryness. The residue was then purified by column chromatography on silica gel using dichloromethane/methanol (90/10: *v*/*v*) as eluent to give the pure products **4a**–**p**.

##### 2,9-*Bis*[(2-dimethylaminoethyl)aminomethyl]-4,7-diphenyl-1,10-phenanthroline (**4a**)

Orange oil (71%). ^1^H NMR (CDCl_3_) δ: 7.8 (s, 2H, H-3 and H-8), 7.76 (s, 2H, H-5 and H-6), 7.55–7.50 (m, 10H, H-phenyl), 4.41 (s, 4H, NCH_2_), 2.91 (t, 4H, *J* = 6.90 Hz, NCH_2_), 2.54 (t, 4H, *J* = 6.90 Hz, NCH_2_), 3.01 (bs, 2H, NH), 2.26 (s, 12H, N(CH_3_)_2_). ^13^C NMR (CDCl_3_): 161.7 (C-2 and C-9), 156.8 (C-4 and C-7), 150.3 (C-1a and C-10a), 139.7 (C-1′), 131.1 (C-3′ and C-5′), 129.9 (C2′and C6′), 129.8 (C-4′), 127.0 (C-4a and C-6a), 124.8 (C-5 and C-6), 123.8 (C-3 and C-8), 60.7 (NCH_2_), 57.8 (NCH_2_), 48.8 (NCH_2_), 47.0 (N(CH_3_)_2_). ESI-MS m/z [M+H]^+^ Calculed for C_34_H_41_N_6_: 533.3393, Found: 533.3382.

##### 2,9-*Bis*[(3-dimethylaminopropyl)aminomethyl]-4,7-diphenyl-1,10-phenanthroline (**4b**)

Orange oil (83%). ^1^H NMR (CDCl_3_) δ: 7.76 (s, 2H, H-3 and H-8), 7.66 (s, 2H, H-5 and H-6), 7.49–7.43 (m, 10H, H-phenyl), 4.32 (s, 4H, 2 NCH_2_), 3.67 (bs, 2H, 2 NH), 2.83 (t, 4H, *J* = 7.20 Hz, 2 NCH_2_), 2.36 (t, 4H, *J* = 7.20 Hz, 2 NCH_2_), 2.19 (s, 12H, 2 N(CH_3_)_2_), 1.78 (qt, 4H, *J* = 7.20 Hz, 2CH_2_). ^13^C NMR (CDCl_3_): 161.3 (C-2 and C-9), 150.3 (C-4 and C-7), 147.4 (C-1a and C-10a), 139.5 (C-1′), 131.5 (C-3′ and C-5′), 129.9 (C-2′, C-6′ and C-4′), 127.0 (C-4a and C-6a), 124.8 (C-5 and C-6), 124.0 (C-3 and C-8), 59.3 (2 NCH_2_), 57.6 (2 NCH_2_), 49.7 (2 NCH_2_), 46.9 (2 N(CH_3_)_2_), 29.4 (2 CH_2_). ESI-MS m/z [M+H]^+^ Calculed for C_36_H_45_N_6_: 561.3706, Found: 561.3705.

##### 2,9-*Bis*[(4-dimethylaminobutyl)aminomethyl]-4,7-diphenyl-1,10-phenanthroline (**4c**)

Orange oil (98%). ^1^H NMR (CDCl_3_) δ: 7.86 (s, 2H, H-3 and H-8), 7.72 (s, 2H, H-5 and H-6), 7.53–7.47 (m, 10H, H-phenyl), 4.35 (s, 4H, 2 NCH_2_), 2.83 (t, 4H, *J* = 6.90 Hz, 2 NCH_2_), 2.29 (t, 4H, *J* = 6.90 Hz, 2 NCH_2_), 2.21 (s, 12H, 2 N(CH_3_)_2_), 1.67–1.53 (m, 8H, 4CH_2_). ^13^C NMR (CDCl_3_): 161.5 (C-2 and C-9), 150.3 (C-4 and C-7), 147.4 (C-1a and C-10a), 139.6 (C-1′), 131.1 (C-3′ and C-5′), 129.9 (C2′ and C6′), 129.8 (C-4′), 127.0 (C-4a and C-6a), 124.8 (C-5 and C-6), 123.8 (C-3 and C-8), 61.1 (2 NCH_2_), 57.7 (2 NCH_2_), 51.4 (2 NCH_2_), 46.8 (2 N(CH_3_)_2_), 29.5 (2 CH_2_), 27.0 (2 CH_2_). ESI-MS m/z [M+H]^+^ Calculed for C_38_H_49_N_6_: 589.4019, Found: 589.4007.

##### 2,9-*Bis*[(5-dimethylaminopentyl)aminomethyl]-4,7-diphenyl-1,10-phenanthroline (**4d**)

Yellow-orange oil (65%). ^1^H NMR (CDCl_3_) δ: 7.81 (s, 2H, H-3 and H-8), 7.72 (s, 2H, H-5 and H-6), 7.56–7.51 (m, 10H, H-phenyl), 4.36 (s, 4H, 2 NCH_2_), 2.83 (t, 4H, *J* = 7.20 Hz, 2 NCH_2_), 2.43 (bs, 2H, 2 NH), 2.27 (t, 4H, *J* = 7.20 Hz, 2 NCH_2_), 2.22 (s, 12H, 2 N(CH_3_)_2_), 1.65 (qt, 4H, *J* = 7.20 Hz, 2CH_2_), 1.51–1.38 (m, 8H, 4 CH_2_). ^13^C NMR (CDCl_3_): 161.3 (C-2 and C-9), 150.3 (C-4 and C-7), 147.4 (C-1a and C-10a), 139.5 (C-1′), 131.1 (C-3′ and C-5′), 129.9 (C-2′, C-6′ and C-4′), 127.0 (C-4a and C-6a), 124.8 (C-5 and C-6), 124.1 (C-3 and C-8), 61.1 (NCH_2_), 57.6 (NCH_2_), 51.4 (NCH_2_), 46.8 (N(CH_3_)_2_), 31.5 (CH_2_), 29.0 (CH_2_), 29.6 (CH_2_). ESI-MS m/z [M+H]^+^ Calculed for C_40_H_54_N_6_: 618.4410, Found: 618.4393.

##### 2,9-*Bis* [2-(4-methylpiperazin-1-yl)ethyl)aminomethyl]-4,7-diphenyl-1,10-phenanthroline (**4e**)

Yellow-orange oil, (68%). 1H NMR (CDCl_3_) δ: 7.81 (s, 2H, H-3 and H-8), 7.77 (s, 2H, H-5 and H-6), 7.56–7.50 (m, 10H, H-phenyl), 4.41 (s, 4H, 2 NCH_2_), 2.91 (t, 4H, *J* = 6.60 Hz, 2 NCH_2_), 2.76 (bs, 2H, 2 NH), 2.61 (t, 4H, *J* = 6.60 Hz, 2 NCH_2_), 2.57–2.36 (m, 16H, 8 NCH_2pip_), 2.27 (s, 6H, 2 NCH_3_). ^13^C NMR (CDCl_3_): 161.9 (C-2 and C-9), 150.3 (C-4 and C-7), 146.9 (C-1a and C-10a), 139.6 (C-1′), 131.1 (C-3′ and C-5′), 129.9 (C2′and C6′), 129.8 (C-4′), 127.0 (C-4a and C-6a), 124.8 (C-5 and C-6), 123.7 (C-3 and C-8), 59.4 (NCH_2_), 57.8 (NCH_2_), 56.5 (NCH_2pip_), 54.7 (NCH_2pip_), 47.9 (NCH_2_), 47.4 (NCH_3_). ESI-MS m/z [M+H]^+^ Calculed for C_40_H_51_N_8_: 643.4236, Found: 643.4229.

##### 2,9-*Bis*[[(3-(4-methylpiperazin-1-yl)propyl)aminomethyl]-4,7-diphenyl-1,10-phenanthroline (**4f**)

Yellow oil (88%). ^1^H NMR (CDCl_3_) δ: 7.79 (s, 2H, H-3 and H-8), 7.69 (s, 2H, H-5 and H-6), 7.54–7.48 (m, 10H, H-phenyl), 4.34 (s, 4H, 2 NCH_2_), 3.02 (bs, 2H, 2 NH), 2.86 (t, 4H, *J* = 6.90 Hz, 2 NCH_2_), 2.49–2.41 (m, 20H, 10 NCH_2_), 2.24 (s, 6H, 2 NCH_3_), 1.82 (qt, 4H, *J* = 6.90 Hz, 2 CH_2_). ^13^C NMR (CDCl_3_): 161.2 (C-2 and C-9), 150.4 (C-4 and C-7), 147.3 (C-1a and C-10a), 139.5 (C-1′), 131.0 (C-3′ and C-5′), 130.3 (C-4′), 129.9 (C-2′ and C-6′), 127.0 (C-4a and C-6a), 124.8 (C-5 and C-6), 124.0 (C-3 and C-8), 58.2 (NCH_2_), 57.4 (NCH_2_), 56.4 (NCH_2pip_), 54.5 (NCH_2pip_), 50.0 (NCH_2_), 47.3 (NCH_3_), 28.4 (CH_2_). ESI-MS m/z [M+H]^+^ Calculed for C_42_H_56_N_8_: 672.4628, Found: 672.4622.

##### 2,9-*Bis*[[(4-(4-methylpiperazin-1-yl)butyl)aminomethyl]-4,7-diphenyl-1,10-phenanthroline (**4g**)

Yellow oil (88%). ^1^H NMR (CDCl_3_) δ: 7.76 (s, 2H, H-3 and H-8), 7.69 (s, 2H, H-5 and H-6), 7.48–7.42 (m, 10H, H-phenyl), 4.32 (s, 4H, 2 NCH_2_), 2.96 (bs, 2H, 2 NH), 2.80 (t, 4H, *J* = 6.60 Hz, 2 NCH_2_), 2.42–2.30 (m, 20H, 2 NCH_2_, 8 NCH_2pip_), 2.23 (s, 6H, 2 NCH_3_), 1.61–1.56 (m, 8H, 4 CH_2_). ^13^C NMR (CDCl_3_): 161.6 (C-2 and C-9), 150.2 (C-4 and C-7), 147.3 (C-1a and C-10a), 139.6 (C-1′), 131.0 (C-3′ and C-5′), 129.9 (C-2′ and C-6′), 129.8 (C-4′), 126.9 (C-4a and C-6a), 124.8 (C-5 and C-6), 123.9 (C-3 and C-8), 59.9 (NCH_2_), 57.6 (NCH_2_), 56.5 (NCH_2pip_), 54.5 (NCH_2pip_), 51.3 (NCH_2_), 47.4 (NCH_3_), 29.6 (CH_2_), 26.1 (CH_2_). ESI-MS m/z [M+H]^+^ Calculed for C_44_H_59_N_8_: 699.4862, Found: 699.4839.

##### 2,9-*Bis* [2-(morpholin-1-yl)ethyl)aminomethyl]-4,7-diphenyl-1,10-phenanthroline (**4h**)

Yellow oil, (73%). ^1^H NMR (CDCl_3_) δ: 7.81 (s, 2H, H-3 and H-8), 7.74 (s, 2H, H-5 and H-6), 7.58–7.44 (m, 10H, H-phenyl), 4.40 (s, 4H, 2 NCH_2_), 3.68 (t, 8H, *J* = 4.30 Hz, 4 OCH_2_), 3.24 (bs, 2H, 2 NH), 2.91 (t, 4H, *J* = 6.60 Hz, 2 NCH_2_), 2.60 (t, 4H, *J* = 6.60 Hz, 2 NCH_2_), 2.47 (t, 8H, *J* = 4.30 Hz, 4 NCH_2morph_). ^13^C NMR (CDCl_3_): 161.6 (C-2 and C-9), 150.4 (C-4 and C-7), 147.4 (C-1a and C-10a), 139.6 (C-1′), 131.0 (C-3′ and C-5′), 129.9 (C2′, C6′ and C-4′), 127.0 (C-4a and C-6a), 124.8 (C-5 and C-6), 123.8 (C-3 and C-8), 68.3 (4 OCH_2_), 59.8 (2 NCH_2_), 57.7 (2 NCH_2_), 55.2 (4 NCH_2morph_), 47.4 (2 NCH_2_). ESI-MS m/z [M+H]^+^ Calculed for C_38_H_45_N_6_O_4_: 617.3604, Found: 617.3596.

##### 2,9-*Bis* [3-(morpholin-1-yl)propyl)aminomethyl]-4,7-diphenyl-1,10-phenanthroline (**4i**)

Yellow oil, (98%). 1H NMR (CDCl_3_) δ: 7.79 (s, 2H, H-3 and H-8), 7.67 (s, 2H, H-5 and H-6), 7.53–7.48 (m, 10H, H-phenyl), 4.34 (s, 4H, 2 NCH_2_), 3.66 (t, 8H, *J* = 4.80 Hz, 4 OCH_2_), 3.51 (bs, 2H, 2 NH), 2.87 (t, 4H, *J* = 6.90 Hz, 2 NCH_2_), 2.48–2.42 (m, 8H, 4 NCH_2morph_), 1.82 (qt, 4H, *J* = 6.90 Hz, 2 CH_2_). ^13^C NMR (CDCl_3_): 161.0 (C-2 and C-9), 150.5 (C-4 and C-7), 147.3 (C-1a and C-10a), 139.4 (C-1′), 131.0 (C-3′ and C-5′), 130.0 (C2′and C6′), 129.9 (C-4′), 127.0 (C-4a and C-6a), 124.9 (C-5 and C-6), 124.1 (C-3 and C-8), 68.3 (2 OCH_2_), 58.6 (2 NCH_2_), 57.4 (2 NCH_2_), 55.1 (4 NCH_2morph_), 49.8 (2 NCH_2_), 28.0 (2 CH_2_). ESI-MS m/z [M+H]^+^ Calculed for C_40_H_53_N_6_O_2_: 645.3917, Found: 645.3912.

##### 2,9-*Bis* [2-(piperidin-1-yl)ethyl)aminomethyl]-4,7-diphenyl-1,10-phenanthroline (**4j**)

Orange oil, (73%). ^1^H NMR (CDCl_3_) δ: 7.79 (s, 2H, H-3 and H-8), 7.74 (s, 2H, H-5 and H-6), 7.53–7.50 (m, 10H, H-phenyl), 4.39 (s, 4H, 2 NCH_2_), 3.35 (bs, 2H, 2 NH), 2.90 (t, 4H, *J* = 6.30 Hz, 2 NCH_2_), 2.56 (t, 4H, *J* = 6.30 Hz, 2 NCH_2_), 2.43–2.39 (m, 8H, 4 NCH_2pip_), 1.58–1.51 (m, 8H, 4 CH_2pip_), 1.43–1.38 (m, 4H, 2 CH_2pip_). ^13^C NMR (CDCl_3_): 161.8 (C-2 and C-9), 150.3 (C-4 and C-7), 147.4 (C-1a and C-10a), 139.6 (C-1′), 131.1 (C-3′ and C-5′), 129.9 (C2′, C6′ and C-4′), 126.9 (C-4a and C-6a), 124.8 (C-5 and C-6), 123.8 (C-3 and C-8), 60.2 (NCH_2_), 57.7 (NCH_2_), 56.2 (NCH_2pip_), 48.1 (NCH_2_), 27.3 (CH_2pip_), 25.8 (CH_2pip_). ESI-MS m/z [M+H]^+^ Calculed for C_40_H_49_N_6_: 613.4019, Found: 613.4020.

##### 2,9-*Bis* [3-(piperidin-1-yl)propyl)aminomethyl]-4,7-diphenyl-1,10-phenanthroline (**4k**)

Yellow-orange oil, (97%). ^1^H NMR (CDCl_3_) δ: 7.76 (s, 2H, H-3 and H-8), 7.71 (s, 2H, H-5 and H-6), 7.50–7.44 (m, 10H, H-phenyl), 4.34 (S, 4H, 2 NCH_2_), 2.82 (t, 4H, *J* = 6.90 Hz, 2 NCH_2_), 2.41–2.30 (m, 12H, 2 NCH_2_ and 4 NCH_2pip_), 1.78 (qt, 4H, *J* = 6.90 Hz, 2 CH_2_), 1.50 (qt, 8H, *J* = 5.40 Hz, 4 CH_2pip_), 1.40–1.36 (m, 4H, 2 CH_2pip_). ^13^C NMR (CDCl_3_): 161.8 (C-2 and C-9), 150.2 (C-4 and C-7), 147.3 (C-1a and C-10a), 139.6 (C-1′), 131.0 (C-3′ and C-5′), 129.8 (C2′and C6′), 129.7 (C-4′), 126.9 (C-4a and C-6a), 124.7 (C-5 and C-6), 123.7 (C-3 and C-8), 59.1 (NCH_2_), 57.8 (NCH_2_), 56.0 (NCH_2pip_), 50.2 (NCH_2_), 28.7 (CH_2_), 27.3 (CH_2pip_), 25.8 (CH_2pip_). ESI-MS m/z [M+H]^+^ Calculed for C_42_H_53_N_6_: 641.4331, Found: 641.4322.

##### 2,9-*Bis* [3-(pyrrolidin-1-yl)propyl)aminomethyl]-4,7-diphenyl-1,10-phenanthroline (**4l**)

Yellow oil, (73%). ^1^H NMR (CDCl_3_) δ: 7.78 (s, 2H, H-3 and H-8), 7.71 (s, 2H, H-5 and H-6), 7.52–7.45 (m, 10H, H-phenyl), 4.34 (s, 4H, 2 NCH_2_), 2.91 (bs, 2H, 2 NH), 2.86 (t, 4H, *J* = 6.90 Hz, 2 NCH_2_), 2.55 (t, 4H, *J* = 6.90 Hz, 2 NCH_2_), 2.48–2.45 (m, 8H, 4 NCH_2pyrr_), 1.82 (qt, 4H, *J* = 6.90 Hz, 2 CH_2_), 1.76–1.71 (m, 8H, 4 CH_2pyr_). ^13^C NMR (CDCl_3_): 161.7 (C-2 and C-9), 150.2 (C-4 and C-7), 147.5 (C-1a and C-10a), 139.6 (C-1′), 131.0 (C-3′ and C-5′), 129.9 (C2′and C6′), 129.8 (C-4′), 126.9 (C-4a and C-6a), 124.8 (C-5 and C-6), 123.8 (C-3 and C-8), 57.8 (2 NCH_2_), 56.1 (2 NCH_2_), 55.6 (4 NCH_2pyrr_), 50.0 (2 NCH_2_), 30.9 (2 CH_2pyrr_), 24.8 (4 CH_2pyrr_). ESI-MS m/z [M+H]^+^ Calculed for C_42_H_49_N_6_: 613.4019, Found: 613.4020.

##### 2,9-*Bis* [2-(pyridin-2-yl)ethyl)aminomethyl]-4,7-diphenyl-1,10-phenanthroline (**4m**)

Orange oil, (97%). ^1^H NMR (CDCl_3_) δ: 8.47 (ddd, 2H, *J* = 5.80, 1.85 and 0.9 Hz, H-6_pyr_), 7.78 (s, 2H, H-3 and H-8), 7.69 (s, 2H, H-5 and H-6), 7.54 (ddd, 2H, *J* = 7.50, 7.10 and 1.85 Hz, H-4_pyr_), 7.51–7.46 (m, 10H, H-phenyl), 7.21 (ddd, 2H, *J* = 7.10, 0.90 and 0.9 Hz, H-3_pyr_), 7.05 (ddd, 2H, *J* = 7.50, 5.80 and 0.9 Hz, H-5_pyr_), 4.40 (s, 4H, 2 NCH_2_), 3.22 (t, 4H, *J* = 6.30 Hz, 2 NCH_2_), 3.09 (t, 4H, *J* = 6.30 Hz 2 CH_2pyr_). ^13^C NMR (CDCl_3_): 161.7 (C-2, C-9 and C-2_pyr_), 150.6 (C-6_pyr_), 150.2 (C-4 and C-7), 147.3 (C-1a and C-10a), 137.7 (C-4_pyr_), 131.1 (C-3′ and C-5′), 129.8 (C2′, C6′), 129.7 (C-4′), 126.9 (C-4a and C-6a), 124.7 (C-5, C-6 and C-3_pyr_), 123.7 (C-3 and C-8), 122.6 (C-5_pyr_), 57.6 (NCH_2_), 50.8 (NCH_2_), 40.0 (CH_2pyr_). ESI-MS m/z [M+H]^+^ Calculed for C_40_H_37_N_6_: 601.3079, Found: 601.3070.

##### 2,9-*Bis* [2-(pyridin-3-yl)ethyl)aminomethyl]-4,7-diphenyl-1,10-phenanthroline (**4n**)

Orange oil, (80%). ^1^H NMR (CDCl_3_) δ: 8.51 (ddd, 2H, *J =* 1.90, 0.90 and 0.9 Hz, 2 H-2_pyr_), 8.41 (dd, 2H, *J =* 5.90 and 1.50 Hz, 2 H-6_pyr_), 7.80 (s, 2H, H-3 and H-8), 7.63 (s, 2H, H-5 and H-6), 7.56 (ddd, 2H, *J* = 7.80, 1.80 and 1.80 Hz, 2 H-4_pyr_), 7.51–7.47 (m, 10H, 10 H-phenyl), 7.17 (ddd, 2H, *J* = 7.80, 5.90 and 0.9 Hz, H-5_pyr_), 4.38 (s, 4H, 2 NCH_2_), 3.30 (bs, 4H, 2 NH), 3.08 (t, 4H, *J* = 6.90 Hz, 2 NCH_2_), 2.93 (t, 4H, *J* = 6.30 Hz, 2 CH_2pyr_). ^13^C NMR (CDCl_3_): 160.9 (C-2 and C-9), 151.6 (C-2_pyr_), 150.5 (C-4 and C-7), 149.0 (C-6_pyr_), 147.3 (C-1a and C-10a), 139.4 (C-1′), 137.6 (C-4_pyr_), 136.8 (C-3_pyr_), 131.0 (C-3′ and C-5′), 129.9 (C2′, C6′ and C-4′), 127.1 (C-4a and C-6a), 124.9 (C-5 and C-6), 124.7 (C-5_pyr_), 124.0 (C-3 and C-8), 57.3 (NCH_2_), 52.2 (NCH_2_), 35.0 (CH_2pyr_). ESI-MS m/z [M+H]^+^ Calculed for C_40_H_37_N_6_: 601.3079, Found: 601.3069.

##### 2,9-*Bis* [2-(pyridin-4-yl)ethyl)aminomethyl]-4,7-diphenyl-1,10-phenanthroline (**4o**)

Orange oil, (98%). ^1^H NMR (CDCl_3_) δ: 8.41 (dd, 4H, *J* = 6.00 and 1.65 Hz, H-2_pyr_ and H-6_pyr_), 7.77 (s, 2H, H-3 and H-8), 7.62 (s, 2H, H-5 and H-6), 7.47–7.43 (m, 10H, H-phenyl), 7.13 (dd, 4H, *J* = 6.00 and 1.65 Hz, H-3_pyr_ and H-5_pyr_), 4.33 (s, 4H, 2 NCH_2_), 3.05 (t, 4H, *J* = 7.20 Hz, 2 NCH_2_), 2.86 (t, 4H, *J* = 6.30 Hz, 2 CH_2pyr_). ^13^C NMR (CDCl_3_): 161.2 (C-2 and C-9), 151.1 (C-2_pyr_ and C-6_pyr_), 150.5 (C-4_pyr_), 150.4 (C-4 and C-7), 147.3 (C-1a and C-10a), 139.4 (C-1′), 131.0 (C-3′ and C-5′), 129.9 (C2′, C6′ and C-4′), 127.0 (C-4a and C-6a), 125.6 (C-3_pyr_ and C-5_pyr_), 124.9 (C-5 and C-6), 123.8 (C-3 and C-8), 57.4 (NCH_2_), 51.4 (NCH_2_), 37.3 (CH_2pyr_). ESI-MS m/z [M+H]^+^ Calculed for C_40_H_37_N_6_: 601.3079, Found: 601.3073.

##### 2,9-*Bis* [3-(pyridin-4-yl)propyl)aminomethyl]-4,7-diphenyl-1,10-phenanthroline (**4p**)

Yellow oil, (94%). ^1^H NMR (CDCl_3_) δ: 8.42 (dd, 4H, *J* = 6.00 and 1.50 Hz, H-2_pyr_ and H-6_pyr_), 7.81 (s, 2H, H-3 and H-8), 7.67 (s, 2H, H-5 and H-6), 7.53–7.49 (m, 10H, H-phenyl), 7.09 (dd, 4H, *J* = 6.00 and 1.50 Hz, H-3_pyr_ and H-5_pyr_), 4.27 (s, 4H, 2 NCH_2_), 3.32 (bs, 2H, 2 NH), 2.76 (t, 4H, J = 6.90 Hz, 2 NCH_2_), 2.66 (t, 4H, *J* = 6.90 Hz, 2 CH_2pyr_), 1.88 (qt, 4H, J = 6.90 Hz, 2 CH_2_). ^13^C NMR (CDCl_3_): 160.9 (C-2 and C-9), 152.5 (C-4_pyr_), 150.9 (C-2_pyr_ and C-6_pyr_), 150.5 (C-4 and C-7), 147.3 (C-1a and C-10a), 139.3 (C-1′), 131.0 (C-3′ and C-5′), 130.0 (C2′ and C6′), 129.9 (C-4′), 127.0 (C-4a and C-6a), 125.3 (C-3_pyr_ and C-5_pyr_), 124.9 (C-5 and C-6), 124.2 (C-3 and C-8), 57.3 (2 NCH_2_), 50.4 (2 NCH_2_), 34.2 (CH_2pyr_), 31.9 (2 CH_2_). ESI-MS m/z [M+H]^+^ Calculed for C_42_H_41_N_6_: 629.3392, Found: 629.3382.

#### 2.1.5. General procedure for 2,9-bis[(substituted-aminomethyl)phenyl]-4,7-diphenyl-1,10-phenanthrolines **1a-p**

To a solution of compounds **4** (0.3 mmol) in isopropanol (11 mL) oxalic acid (2.4 mmol, 8 eq.) was added. The reaction mixture was heated under reflux for 30 min. The precipitate was filtered, washed with isopropanol then with diethyl ether and dried under reduced pressure to give the ammonium oxalate salts **1**.

### 2.2. Biological Evaluation

#### 2.2.1. In Vitro Antiplasmodial Activity

The in vitro antiplasmodial activities were tested over concentrations ranging from 39 nM to 40 µM against culture-adapted *Plasmodium falciparum* reference strains 3D7 and W2. The former strain is susceptible to chloroquine (CQ) but displays a decreased susceptibility to mefloquine (MQ); the latter is considered to be resistant to CQ. These two strains are obtained from the collection of the National Museum of Natural History (Paris, France). The parasites were cultivated in RPMI medium (Sigma Aldrich, Lyon, France) supplemented with 0.5% Albumax I (Life Technologies Corporation, Paisley, UK), hypoxanthine (Sigma Aldrich), and gentamicin (Sigma Aldrich) with human erythrocytes and were incubated at 37 °C in a candle jar, as described previously [39]. The *P. falciparum* drug susceptibility test was carried out in 96-well flat bottom sterile plates in a final volume of 250 µL. After 48 h incubation period with the drugs, quantities of DNA in treated and control cultures of parasites in human erythrocytes were quantified using the SYBR Green I (Sigma Aldrich) fluorescence-based method [40,41]. Briefly, after incubation, plates were frozen at -20 °C until use. Plates were then thawed for 2 h at room temperature, and 100 µL of each homogenized culture was transferred to a well of a 96-well flat bottom sterile black plate (Nunc, Inc.) that contained 100 µL of the SYBR Green I lysis buffer (2xSYBR Green, 20 mM Tris base pH 7.5, 5 mM EDTA, 0.008% *w*/*v* saponin, 0.08% *w*/*v* Triton X-100). Negative controls treated with solvent (typically DMSO or H_2_O), and positive controls (CQ and MQ) were added to each set of experiments. Plates were incubated for 1 h at room temperature and then read on a fluorescence plate reader (Tecan, Austria) using excitation and emission wavelengths of 485 and 535 nm, respectively. The concentrations at which the screening drug or antimalarial is capable of inhibiting 50% of parasitic growth (IC_50_) are calculated from a sigmoid inhibition model Emax with an estimate of IC_50_ by non-linear regression (IC Estimator version 1.2) and are reported as means calculated from three independent experiments [42].

#### 2.2.2. In Vitro Antileishmanial Activity

*L. donovani* (MHOM/IN/00/DEVI) used in this study was provided by the CNR Leishmania (Montpellier, France). The effects of the tested compounds on the growth of *L. donovani* (MHOM/IN/00/DEVI) promastigotes were assessed by MTT assay [43]. Briefly, promastigotes in log-phase in Schneider’s medium supplemented with 20% fetal calf serum (FCS), 2 mM L-glutamine and antibiotics (100 U/mL penicillin and 100 µg/mL streptomycin), were incubated at an average density of 10^6^ parasites/mL in sterile 96-well plates with various concentrations of compounds previously dissolved in DMSO (final concentration less than 0.5% *v*/*v*), in duplicate. Appropriate controls treated with DMSO, pentamidine, and amphotericin B (reference drugs purchased from Sigma Aldrich) were added to each set of experiments. Duplicate assays were performed for each sample. After the 72 h incubation period at 27 °C, parasite metabolic activity was determined. Each well was microscopically examined for precipitate formation. To each well was added 10 µL of 10 mg/mL MTT [3-(4,5-dimethylthiazol-2-yl)-2,5-diphenyltetrazolium bromide)] solution followed by 4 h incubation time. The enzyme reaction was stopped by addition of 100 µL of 50% isopropanol/10% sodium dodecyl sulfate [44]. Plates were vigorously shaken (300 rpm) for 10 min, and the absorbance was measured at 570 nm with 630 nm as reference wavelength in a BIOTEK ELx808 Absorbance Microplate Reader (Agilent Technologies, Les Ulis, France). The IC_50_ was defined as the concentration of drug required to inhibit by 50% of the metabolic activity of *L. donovani* promastigotes compared to the control. IC_50_ of the parasite’s growth (half maximal inhibitory concentration or IC_50_ values) were then calculated from the obtained experimental results using a previously described regression program [42]. IC_50_ values were calculated from three independent experiments.

#### 2.2.3. In Vitro Antitrypanosomal Activity

The effects of the tested compounds on the growth of *T. brucei brucei* were assessed using an Alamar Blue^®^ assay described by Räz et al. [45] *T. brucei brucei* AnTat 1.9 (IMTA, Antwerpen, Belgium) was cultured in MEM with Earle’s salts, supplemented according to the protocol of Baltz et al. [46] with the following modifications: 0.5 mM mercaptoethanol (Sigma Aldrich), 1.5 mM L-cysteine (Sigma Aldrich), 0.05 mM bathocuproine sulfate (Sigma Aldrich), and 20% heat-inactivated horse serum (Gibco, France) at 37 °C and 5% CO_2_. Samples were incubated at an average density of 2000 parasites/well in sterile 96-wells plates (Fisher, France) with various concentrations of compounds dissolved in 0.9% NaCl. All doses were tested in duplicate. Appropriate controls treated with solvents 0.9% NaCl or DMSO or with suramin, pentamidine, eflornithine, and fexinidazole (reference drugs purchased from Sigma Aldrich and Fluorochem, UK) were added to each set of experiments. After 69 h incubation period at 37 °C, 10 µL of the viability marker Alamar Blue (Fisher) was added to each well, and the plates were incubated for 5 h. The plates were read in a PerkinElmer ENSPIRE (Germany) microplate reader using an excitation wavelength of 530 nm and an emission wavelength of 590 nm. The IC_50_ was defined as the concentration of drug necessary to inhibit by 50% the activity of *T. brucei brucei* compared to the control. IC_50_ values were calculated using a nonlinear regression analysis of dose-response curves performed using GraphPad Prism software (GraphPad Software, San Diego, CA, USA). IC_50_ values were calculated from three independent experiments.

#### 2.2.4. Cytotoxicity Evaluation

A cytotoxicity evaluation was performed using the method reported by Mosmann [43] with slight modifications to determine the cytotoxic concentrations 50% (CC_50_) and using doxorubicin as a cytotoxic reference compound. These assays were performed in human HepG2 cells purchased from ATCC (ref HB-8065). These cells are a commonly used human hepatocarcinoma-derived cell line that has characteristics similar to those of primary hepatocytes. These cells express many hepatocyte-specific metabolic enzymes, thus enabling the cytotoxicity of tested product metabolites to be evaluated. Briefly, cells in 100 µL of complete RPMI medium, (RPMI supplemented with 10% FCS, 1% L-glutamine (200 mM), penicillin (100 U/mL), and streptomycin (100 µg/mL)) were inoculated at 37 °C into each well of 96-well plates in a humidified chamber in 6% CO_2_. After 24 h, 100 µL of medium with test compound at various concentrations dissolved in DMSO (final concentration less than 0.5% *v*/*v*) were added, and the plates were incubated for 72 h at 37 °C. Duplicate assays were performed for each sample. Each well was microscopically examined for precipitate formation before the medium was aspirated from the wells. After aspiration, 100 µL of MTT solution (0.5 mg/mL in medium without FCS) were then added to each well. Cells were incubated for 2 h at 37 °C. The MTT solution was removed, and DMSO (100 µL) was added to dissolve the resulting blue formazan crystals. Plates were shaken vigorously (300 rpm) for 5 min. The absorbance was measured at 570 nm with 630 nm as reference wavelength in a BIO-TEK ELx808 Absorbance Microplate Reader. DMSO was used as blank and doxorubicin (Sigma Aldrich) as positive control. Cell viability was calculated as percentage of control (cells incubated without compound). The CC_50_ was determined from the dose-response curve using TableCurve 2D V5.0 software (Systat Software, Palo Alto, CA, USA).

### 2.3. FRET Melting Experiments

The bioactive compounds **1a**–**p** were selected for the subsequent FRET melting experiments. These were performed with dual-labeled oligonucleotides mimicking the *Plasmodium* telomeric sequences FPf1T [FAM-5′(GGGTTTA)3-GGG3′-TAMRA] and FPf8T [FAM-5′(GGGTTCA)3GGG3′-TAMRA], the Trypanosoma 9 and 11 chromosomic sequence FTrypBT (also named FEBR1T) [FAM-5′GGGCAGGGGGTGATGGGGAGGAGCCAGGG3′-TAMRA], the human telomeric sequence F21T [FAM-(GGGTTA)3-GGG3′-TAMRA] and the human duplex sequence FdxT [FAM5′-TATAGCTATA-hexaethyleneglycol-TATAGCTATA3′-TAMRA] [27,31,47]. The oligonucleotides were pre-folded in 10 mM lithium cacodylate buffer (pH 7.2), with 10 mM KCl and 90 mM LiCl (K^+^ condition). The FAM emissions were recorded at 516 nm using a 492-nm excitation wavelength in the absence and presence of a single compound as a function of temperature (25 to 95 °C) in 96-well microplates using a Stratagene MX3000P real-time PCR device at a rate of 1 °C∙min-1. Data were normalized between 0 and 1, and the required temperature for half-denaturation of oligonucleotides corresponding to the emission value of 0.5 was taken as the Tm. Each experiment was performed in duplicate with 0.2 µM of labeled oligonucleotide and 2 µM of compound under K^+^ condition. For each compound, three independent experiments were carried out.

### 2.4. Native Electrospray Mass Spectrometry and Circular Dichroism

#### 2.4.1. Samples

Oligonucleotides were purchased in the lyophilized form from Eurogentec (RP-cartridge purification), and dissolved at 1 mM in pure water. TMAA (1 M) was purchased from Santa Cruz Biotechnology and potassium chloride (purity > 99.99%) from Sigma Aldrich (Sigma Aldrich, Saint-Quentin-Fallavier, France). The sample solutions contained 10 µM oligonucleotide and 10 (for CD only) or 20 µM ligand, in a 100 mM TMAA, 1mM KCl buffer. The solutions were incubated overnight at 4 °C before analysis.

#### 2.4.2. Native Mass Electrospray Spectrometry

The sample solutions were injected at a 10 µM strand concentration on an Agilent 6560 DTIMS-Q-TOF instrument (Agilent Technologies, Santa Clara, CA, USA), equipped with a dual-ESI source operated in the negative ion mode in soft conditions [48]. The following parameters were used: fragmentor 320 V, trap fill time 1000 µs, trap release time 200 µs, trap entrance grid delta 2 V. The drift tube was filled with helium and the pressure was fixed at 3.89 ± 0.01 Torr (measured by a capacitance diaphragm gauge CDG-500, Agilent Technologies). 

The MS data were extracted using the IM-MS Browser (v. B.08.00; Agilent Technologies). The peak area intensity of each species at the 5- charge state was extracted from their corresponding m/z range (encompassing potassium adducts), and baseline subtracted. The concentrations of all species (free DNA, free ligands, and complexes) and corresponding dissociation constants were determined as previously described [49,50].

#### 2.4.3. Circular Dichroism

CD experiments were performed with a JASCO J-1500 spectropolarimeter using quartz cells of 2 mm path length. The scans were recorded at 22 °C, from 220 to 350 nm with the following parameters: 1.0 nm data pitch, 2 nm bandwidth, 0.5 s response, 50 nm/min scanning speed; they are the result of three accumulations. The CD data were blank-subtracted, baseline subtracted and normalized to molar dichroic absorption as previously described [49,50].

## 3. Results

### 3.1. Chemistry

**Scheme 1:**
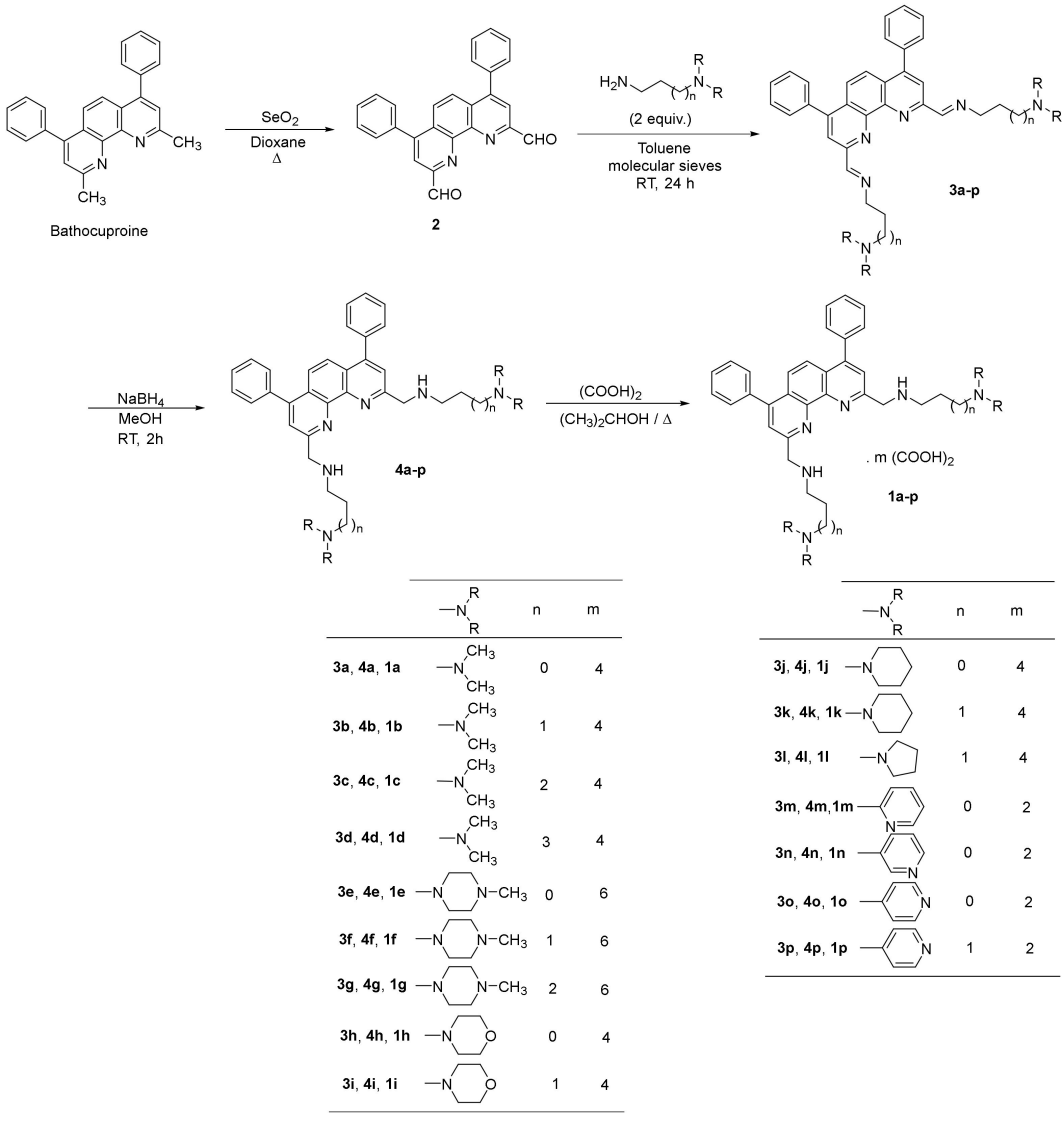
General procedure for the preparation of target compounds **1a**–**p**.

The reported 2,9-bis[(substituted-aminomethyl)]-4,7-phenyl-1,10-phenanthroline derivatives **1a**–**p** were synthesized starting from the commercially available 2,9-dimethyl-4,7-di-phenyl-1,10-phenanthroline, also called bathocuproine (Scheme 1). First, dialdehyde **2** was obtained by oxidation of the bathocuproine using selenium dioxide (SeO_2_) [51]. Reaction of primary substituted alkylaminoalkylamines with **2** gave the 2,9-bis[(substituted-iminomethyl)phenyl]-4,7-diphenyl-1,10-phenanthrolines **3a**–**p**, which were reduced into the 2,9-bis[(substituted-aminomethyl)phenyl]-4,7-diphenyl-1,10-phenanthrolines **4a**–**p** using sodium borohydride in methanol as previously described [27,29,31]. These phenanthroline compounds **4a**–**p** were then converted into their ammonium oxalate salts **1a**–**p** by treatment with oxalic acid in refluxing isopropanol. Table 1 summarizes the physical properties of the ammonium oxalates **1a**–**p**.

### 3.2. Biological Evaluation

#### 3.2.1. In Vitro Antimalarial Activity

All the new phenanthroline derivatives **1a**–**p** were evaluated for their antimalarial activity in vitro by incubation with *P. falciparum* CQ-resistant strain W2 (IC_50_ CQ = 0.40 μM, IC_50_ MQ = 0.016 μM) and the strain 3D7, which is CQ-sensitive and which has decreased sensitivity to MQ (IC_50_ CQ = 0.11 μM, MQ = 0.06 μM) (Table 2). As shown in Table 2, these new phenanthroline derivatives **1** showed IC_50_ values between 0.03 and 11.27 μM against W2, and between 0.47 and 21.19 μM against the 3D7 *P. falciparum* strains.

In terms of structure-activities relationships, the influence of the length of the carbon chain between the two amino functions of the side chain, and also the nature of the terminal amine group, were discussed. Against the *P. falciparum* CQ-resistant strain W2, compounds **1c**, **1i** and **1l** bearing dimethylaminobutylaminomethyl, morpholinopropylaminomethyl, or pyrrolidinopropylaminomethyl side chains at positions 2 and 9 of the phenanthroline moiety were found to be the most active compounds with an IC_50_ of 0.03, 0.04 and 0.03 μM, respectively, in the same range as the one observed for mefloquine (IC_50_ = 0.016 μM). Concerning these phenanthrolines substituted by aminoethylaminomethyl side chains, derivative **1h** bearing morpholinoethylaminomethyl side chains displayed better activity than its three other analogues (IC_50_ = 0.17 μM for **1h** versus IC_50_ = 0.92–2.60 μM for **1a**, **1e** and **1j**). In comparison, when we replaced the aminoethylaminomethyl side functions by aminopropylaminomethyl side chains (compounds **1b**, **1f**, **1i**, **1k** and **1l**), the disubstituted phenanthrolines **1i** and **1l** bearing morpholino C3 aminomethyl or pyrrolidino C3 aminomethyl side chains showed the best antimalarial activity, i.e., IC_50_ = 0.04 μM for **1i** and 0.03 μM for **1l** versus 11.27 μM for their analog **1f** bearing methylpiperazinyl C3 aminomethyl side chains). In the sub-series of the phenanthrolines **1a**–**d** substituted by dimethylaminoalkylaminomethyl side chains, compound **1c** with butyl chains exhibited a better antimalarial activity than its di-substituted ethyl, propyl, or pentyl analogues **1a-b** and **1d** (IC_50_ = 0.03 μM for **1c** versus IC_50_ = 1.00–6.13 μM for the other ones). In the pyridinylethylaminomethyl substituted bisphenanthroline series (compounds **1m**–**o**), derivative **1m** containing (pyridin-2-yl)ethyl)aminomethyl chains was found more active (up to 36.3 or 26.7 times) against the *P. falciparum* W2 strain than its counterpart compounds **1n** and **1o** with (pyridin-3-yl)ethyl)aminomethyl or (pyridin-4-yl)ethyl)aminomethyl chains (IC_50_ = 0.07 μM for **1m** versus IC_50_ = 2.54 and 1.87 μM for **1n** and **1o**). Moreover, the substitution by (pyridin-4-yl)propyl)aminomethyl chains side chains (compound **1p**) showed a better antimalarial activity against the W2 strain than its homologue substituted at positions 2 and 9 by (pyridin-4-yl)ethyl)aminomethyl chains (compound **1o**); i.e., IC_50_ = 0.145 μM for **1p** versus 1.87 μM for **1o**.

Among the newly reported di-substituted(aminoethylaminomethyl)phenanthroline derivatives **1**, compound **1h** with morpholinoethylaminomethyl side chains exhibited the most potent activity against the 3D7 strain with an IC_50_ of 0.67 μM but slightly more active than those observed for the other bis-aminoethylaminomethyl substituted compounds **1a**, **1e**, and **1j** (IC_50_ = 1.59–2.16 μM). However, this phenanthroline derivative **1h** was noticed less active than the two reference drugs, CQ and MQ, which had IC_50_ values of 0.11 and 0.06 μM, respectively. Moreover, the influence of the length of the carbon chain in the aminoalkylaminomethyl side chains for this phenanthroline series seems also to be detrimental: a longer alkyl chain (aminopropylamine chain to aminopentylamine chain) in comparison with an aminoethylamine chain led to a decreased in the antiplasmodial activity and this whatever the terminal amine function (dimethylamine or methylpiperazine moities) of the side chain, (for example; IC_50_ = 1.80 µM for **1a** vs. 21.19, 10.31 and 4.79 µM for **1b**, **1c,** and **1d**, respectively). In the pyridinylethylaminomethyl substituted bisphenanthroline series (compounds **1m**–**o**), whatever the position of the pyridine moiety on the pyridinylethylaminomethyl side chain, compounds **1m**–**o** showed similar range of antiparasitic activities against the 3D7 strain (IC_50_ = 0.47–0.73 μM).

#### 3.2.2. In Vitro Antileishmanial Activity against Promastigote Forms

To better understand the pharmacological profile of our new phenanthroline heterocycles **1**, some additional antiparasitic analyses were also performed. Notably, *P. falciparum* belongs to the coccidian protozoan parasite family. Therefore, in vitro activity against flagellate protozoan parasite *L. donovani* was evaluated (Table 2). The reference drugs amphotericin B and pentamidine had IC_50_ values of 0.10 µM and 5.50 µM, respectively, against *L. donovani.* Only the phenanthrolines **1h**, **1j**, **1n** and **1o** were found active against the promastigote forms of L. donovani with IC_50_ from 2.52 to 4.50 μM. These biological results show that the activity of these derivatives appears to be related to the presence of an ethyl group substituted by a nitrogen membrered heterocycle, such as morpholine, piperidine or pyridine, i.e., compounds **1h**, **1j**, **1n** and **1o,** respectively. Unfortunately, none of all of the other compounds **1** showed any anti-leishmanial activity in vitro (all IC_50_ values > 12.5 μM).

#### 3.2.3. In Vitro Activity against *Trypanosoma Brucei Brucei*

These novel synthesized aza heterocyclic compounds **1a**–**p** were then evaluated against *T. brucei brucei*. In addition, pentamidine, suramine, fexinidazole, and eflornithine were used here as reference compounds. The screening data are presented in Table 2. All phenanthrolines **1a**–**p** were active against *T. brucei brucei* with IC_50_ values ranging from 0.20 to 1.42 μM; and most of them showed an antiparasitic activity around the 1 μM. In terms of structure-activities relationships, an increase of the alkyl chain in the aminoalkylaminomethyl side chains seems to show a slight decrease in the antitrypanosomal activity, i.e., IC_50_ = 0.45 μM for **1e** versus 0.97 and 0.96 μM for **1f** and **1g**, respectively. In the pyridinylethylaminomethyl substituted bisphenanthroline series (compounds **1m**–**o**), phenanthroline **1m** containing (pyridin-4-yl)ethyl)aminomethyl chains exhibited a better antitrypanosomal activity (IC_50_ = 0.21 μM) than its homologues bearing (pyridin-2-yl)ethyl)aminomethyl or (pyridin-3-yl)ethyl)aminomethyl side chains (compounds **1m** and **1n**) for which IC_50_ were noticed at 0.63 and 0.43 μM, respectively.

#### 3.2.4. Cytotoxicity and Selectivity Index

To assess selectivity of action, the cytotoxicities of these newly synthesized antiparasitic phenanthroline compounds **1** were evaluated in vitro in the human cell line HepG2 (Table 2), which is a commonly used human-derived hepatocarcinoma cell line, and express many hepatocyte-specific metabolic enzymes. The goal of this assay was to evaluate the impact of metabolic activation of the tested phenanthrolines on cell viability [52,53]. The cytotoxic concentrations 50% (CC_50_) were determined, and selectivity indexes (SI), defined as the ratios of cytotoxic to antiparasitic activities (SI = CC_50_/IC_50_) were calculated. The results of cytotoxicity assays and the associated SI values are presented in Table 3. Most of these phenanthrolines **1** that were found active against the various parasites showed significant cytotoxicity against the HepG2 cells with CC_50_ values ranging from 1.64 to 15.17 µM. Concerning the W2 strain, the calculated SIs were noticed between 0.76 and 505.7. For the CQ-sensitive strain 3D7, the SIs were found from 0.28 to 26.18. Analyses of these SI values led us to identify the phenanthrolines **1l** and **1c** as very interesting compounds with SI of 505.7 and 347.67 for the W2 strain, respectively. In addition, the derivative **1i** also showed promising SI value of 157.25 for this CQ-resistant strain W2. In addition, this SI led to the identification of compound **1o** with SI of 7.1 on L. donovani. Against the T. brucei brucei strain, 2,9-bis [2-(pyridin-4-yl)ethyl)aminomethyl]-4,7-diphenyl-1,10-phenanthroline **1o** had SI of 91.0. These promising SI values could indicate that these new nitrogen heterocyclic compounds warrant further investigations into their potential use as antiparasitic drugs.

### 3.3. FRET Melting Experiments

As the telomeres of the parasites *P. falciparum* and *Trypanosoma* could be considered to be potential targets of this kind of nitrogen heterocyclic derivatives [31,36,37,38], we have also investigated the stabilization of the *P. falciparum* telomeric or *T. brucei brucei* chromosomic G-quadruplexes by our pharmacological active derivatives **1** using a FRET melting assays. We used a FRET melting assay to determine the degree to which the new phenanthroline derivatives stabilize the G-quadruplexes formed by oligonucleotides with *P. falciparum* or *T. brucei brucei* as well as human telomeric sequences. For this purpose, we used two fluorescently labeled *P. falciparum* telomeric and one *T. brucei brucei* chromosomic sequences (FPf1T, FPf8T and FtrypBT) and one human telomeric sequence (F21T).

To probe the G4 selectivity of our ligands **1a**–**p** over duplex DNA, a FRET melting assay was performed using a duplex control sequence, FdxT. For comparison, we evaluated reference G4 ligand PhenDC3 and the antimalarial reference drugs CQ and MQ. To enable comparison of selectivities, we calculated the difference (ΔTm) between the Tm of the G-quadruplex formed by FPf1T, FPf8T, FtrypBT (FEBR1T), F21T or FdxT in the presence or absence of each selected compound. These ΔTm values are presented in Table 4. For these selected compounds **1a**–**p**, the ΔTm values ranged from 1.0 to 20.5 °C at 2 µM ligand concentration.

The stabilization of our compounds was also investigated against the fluorescently labeled human telomeric sequence F21T. To probe the G4 selectivity of our heterocyclic ligands **1** over duplex DNA, a FRET melting assay was performed using a duplex control sequence, FdxT (Table 4).

The best ligands which stabilize all the four G-quadruplexes sequences were compounds **1d**, **1g** and **1l** (Table 4). The best di-nitrogen heterocyclic ligand which stabilize all the three parasitic (FPf1T, FPf8T, FtrypBT) and the human F21T G-quadruplexes sequences was the 2,9-bis[(5-dimethylaminopentyl)aminomethyl]-4,7-diphenyl-1,10-phenanthroline **1d** with ΔTm values ranging from 14.7 to 20.5 °C. In each sub-series, it could be observed that the best phenanthroline heterocyclic compounds **1** which exhibited an interesting stabilization profile were those substituted by the longest aminoalkylaminomethyl side chains. For example, for each G-quadruplex sequence, the substituted diphenylphenanthroline ligand **1d** bearing (dimethylaminopentyl)aminomethyl side chains exhibited the best strong stabilization profile in comparison with their (dimethylaminobutyl)aminomethyl or (dimethylaminopropyl)aminomethyl or (dimethylaminoethyl)aminomethyl substituted homologues **1a-c**; these latter being least stabilizing ligands. The same observations could also be noticed for derivative **1g** versus **1e**–**f**; **1i** versus **1h**; and also compound **1k** versus **1j**. Concerning the position of the substitution of the pyridine group on the pyridinylalkylaminomethyl chains (compounds **1m**–**p**), we cannot really conclude in terms of structure-activity relationships.

**Figure 2:**
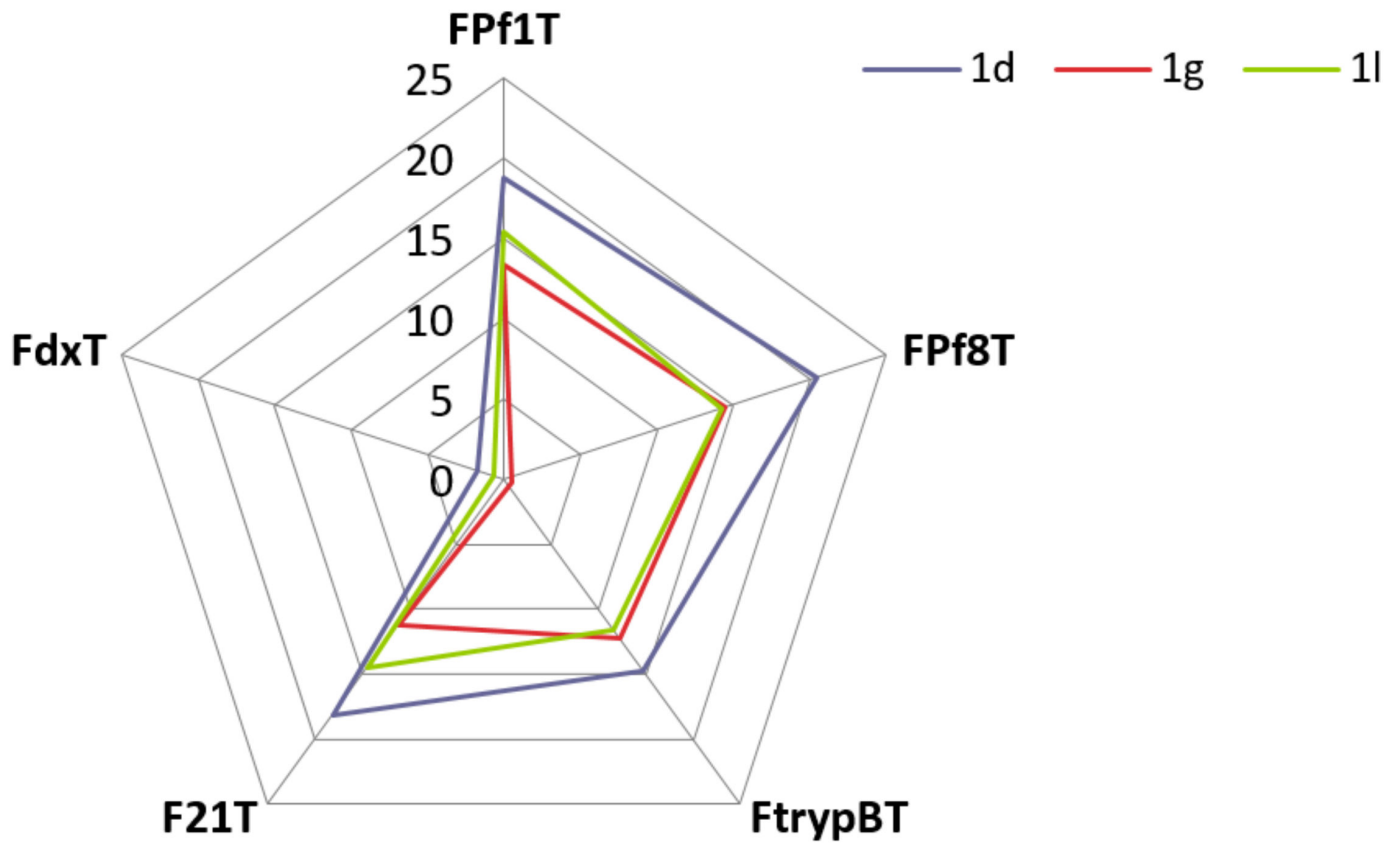
Stabilization specificity profile of **1d**, **1g,** and **1l** (2 µM) toward various G4 oligonucleotides. The difference in Tm in the presence and absence of **1d**, **1g,** and **1l**, ∆Tm, in °C is plotted for each sequence quadruplexes and one duplex (FdxT) were tested.

A radar plot shown in Figure 2 show that compounds **1d**, **1g,** and **1l** are able to stabilize most of all the G4 forming sequences.

From a general point of view, all our ligands **1a**–**p** were found to show lower stabilization than the reference PhenDC3 ligand. FRET assays showed there was no binding to duplex DNA sequence.

### 3.4. Native Electrospray Mass Spectrometry and Circular Dichroism

Native mass spectrometry is the technique of choice to determine the binding affinity and stoichiometry of small molecules with oligonucleotides [54]. Here we performed native ESI-MS experiments with the ligands **1c**, **1d**, **1l** and **1m** and the G4-forming oligonucleotides Pf1 (d[(G_3_T_3_A)_3_G_3_]), Pf8 (d[(G_3_T_2_CA)_3_G_3_]) and the human telomeric sequence 24TTG (Table 5). Two non-G4 forming controls were also used: the duplex-forming sequence DK66 (d[(CG)_2_A_2_T_2_(CG)_2_]) and a G-rich unfolded single-stranded sequence, 24nonG4 (d[TG_3_ATGCGACA(GA)_2_G_2_ACG_3_A]). All tested structures contain 24 nucleotides.

Pf1 and Pf8 are the same *Plasmodium* telomeric sequences used in the FRET meting experiments. Their CD signature suggests the formation of hybrid conformers (positive band at 290 nm, shouldering at 270 nm; Figure S1) 4TTG (d[T_2_G_3_(T_2_AG_3_)_3_A]) is a variant of the human telomeric sequence (similar to the 21T sequence used in the FRET melting experiments) whose hybrid structure in native MS conditions is known [55], and was verified here by CD (Figure S1). UV-melting experiments indicate that these three G4-forming oligonucleotides form G4s of similar stabilities (T_m_ = 38.2, 38.7 and 40.3 °C, respectively). They are fully folded at 22 °C, in our mass spectrometry buffer (folded fraction *θ*~1.0; Figure S2). Native MS spectra confirms the quantitative formation of G4 conformers binding two K^+^, with small amounts of 1K^+^ complexes detected in the case of Pf1 (Figure S3). 

**Figure 3:**
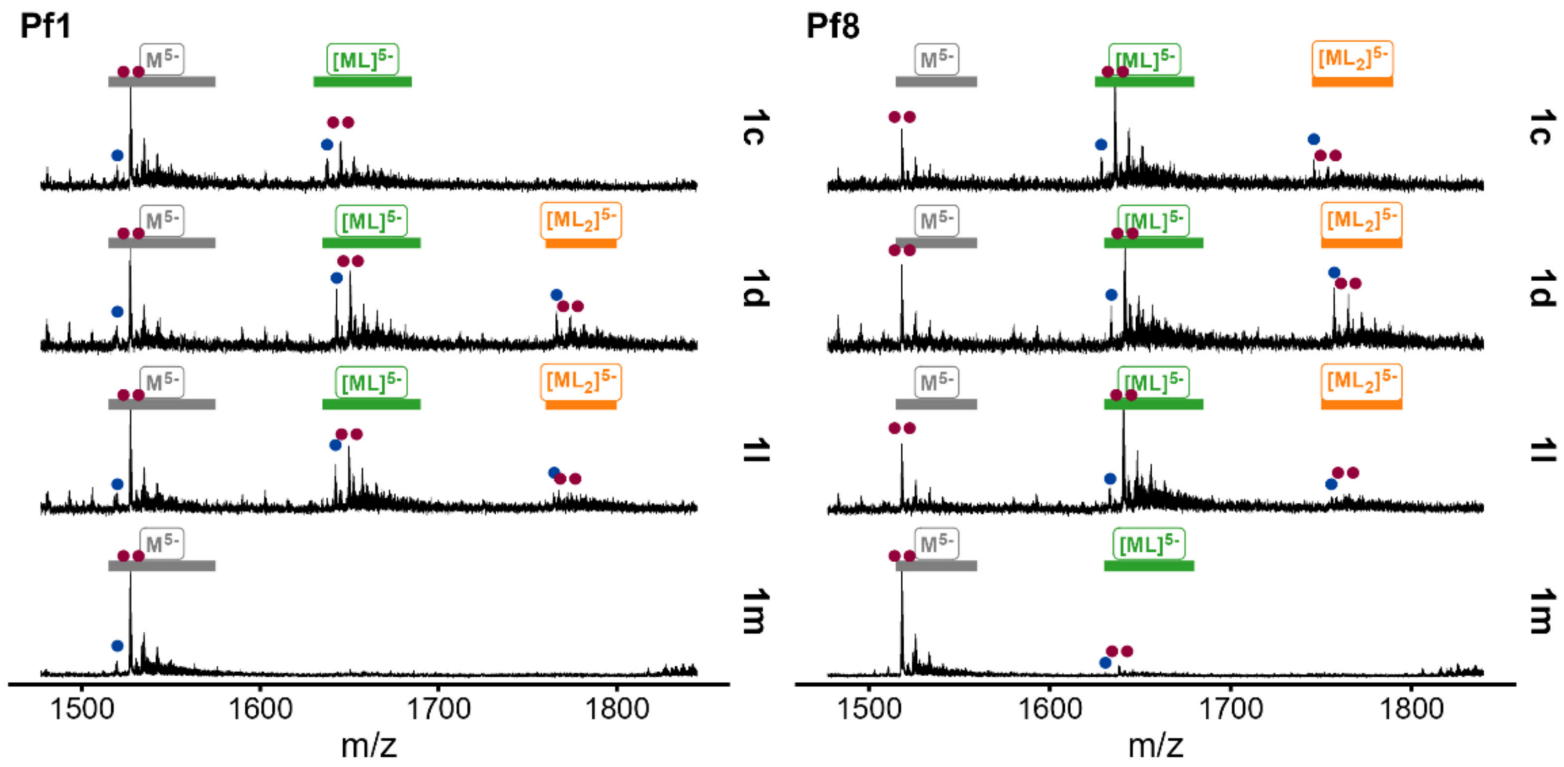
Native ESI-MS spectra showing the 5- charge state species (M: monomer). Free DNA (gray), 1:1 complexes (green) and 2:1 complexes (orange) are annotated on the spectra. Potassium cation stoichiometries are indicated with • (1K^+^) and •• (2K^+^).

**1m** does not bind any of the assayed oligonucleotides (including controls), expect for the oligonucleotide Pf8 that it very weakly bound (Figure 3 and Figure S3). This is consistent with the absence of stabilization observed by FRET melting. 

**1c, 1d**, and **1l** bind moderately to all three G4-forming oligonucleotides (Figure 3 and Figure S3, Table 3). Affinities for any given sequence are similar across these three ligands, with **1c** being somewhat less affine than **1d** and **1l**. Both 1:1 and 2:1 ligand:DNA complexes are formed, with the exception of **1c** that only forms 1:1 complexes with Pf1. The tightest binding occurs with Pf8 (K_d1_ = 2–3 µM and K_d2_ = 16–59 µM). Pf1 is bound with lower affinities (K_d1_ = 8–23 µM and K_d2_ = 24–37 µM). The amount of bound 24TTG is even lower, yet 2:1 complexes can still be observed, highlighting the presence of two weak binding sites of relatively close affinities (K_d1_ = 34–44 µM and K_d2_ = 18–62 µM).

Remarkably, the K^+^ adduct distribution is altered upon ligand binding for all these complexes (Figure 3 and Figure S3). Specifically, the distribution shifts toward the 1K^+^ species, hinting at the formation of two-tetrad conformers. It is more evident for Pf1 than Pf8, consistent with the former already forming a small amount of 1K^+^ species in absence of ligand. Binding of a second ligand further shifts the equilibrium toward the 1K^+^ species. The CD spectra shift toward more antiparallel signatures (positive band at 290 nm, negative band at 260 nm) with increasing ligand concentrations (Figure S1). This is consistent with the formation of two-tetrad, intramolecular G4s coordinating potassium that usually adopt an antiparallel topology [55,56]. Alternatively, this might indicate intercalation of the ligand as recently observed [57]. 

Small amounts of 2:1 complexes with 24TTG devoid of potassium adduct are observed. **1c**, **1d**, and **1l** may therefore promote the unfolding of 24TTG, although to a limited extent. This is supported by their binding to the G-rich non-G4 24nonG4 oligonucleotide with dissociation constants in the micromolar range (K_d1_ = 8–11 µM). Complexation of 24nonG4 does not lead to a shift to larger K^+^ stoichiometries, which could have indicated folding into G4 conformers. CD spectra show that the conformation of 24nonG4 is somewhat altered upon ligand binding, but does not support G4 formation either. These ligands are therefore likely to be able to bind to non-G4 conformations of G-rich oligonucleotides.

Finally, **1c, 1d**, and **1l** form weak 1:1 complexes with the duplex control DK66 (K_d1_ = 53—110 µM), suggesting that they may also act as low-affinity intercalators in genomic DNA.

## 4. Conclusions

In this work, we described the design, synthesis, antiprotozoal activities, and the in vitro cytotoxicity toward human cells of a novel series of 2,9-*bis*[(substituted-aminomethyl)]-4,7-phenyl-1,10-phenanthroline derivatives. These new phenanthrolines were tested for their in vitro antiprotozoal activity toward the CQ-resistant W2 and CQ-sensitive 3D7 *P. falciparum* strains, the promastigote form of *L. donovani*, and also toward a *T. brucei brucei* strain. In addition, the in vitro cytotoxicity of these new nitrogen heterocyclic compounds was assessed on the human HepG2 cell line. Among these novel synthesized nitrogen heterocyclic molecules, a few of them were identified as interesting potential in vitro antiplasmodial leads with IC_50_ ranging from 0.03 to 0.73 µM on the W2 and 3D7 strains of *P. falciparum*. Interestingly, the 2,9-*bis* [3-(pyrrolidin-1-yl)propyl)aminomethyl]-4,7-diphenyl-1,10-phenanthroline **1l** was identified as the most potent and promising antimalarial candidate with a ratio of cytotoxic to antiparasitic activities of 500 against the *P. falciparum* CQ-resistant strain W2. Four substituted phenanthrolines showed activity against the promastigote forms of *L. donovani* with IC_50_ values ranging from 2.52 to 4.50 µM. Moreover, the antiprotozoal activity spectrum of our new synthesized phenanthrolines using a *T. brucei brucei* strain revealed IC_50_ values ranging from 0.20 to 1.42 µM, which warrant further investigations. The 2,9-*bis* [2-(pyridin-4-yl)ethyl)aminomethyl]-4,7-diphenyl-1,10-phenanthroline **1o** was also identified as the most potent trypanosomal candidate with SI of 91 on *Trypanosoma brucei brucei* strain. Structure-activity relationships of these new synthetic compounds are also discussed, as well as their relative ability to target *P. falciparum* or *Trypanosoma* telomeres as a hypothetical mechanism of action and a potential way to escape the efflux resistance developed by parasites toward quinoline drugs. Thus, as the telomeres of the parasites could constitute interesting targets, we also investigated the possibility of targeting *Plasmodium* telomeres or *Trypanosoma* chromosomes by stabilizing the *Plasmodium* or *Trypanosoma* G-quadruplexes sequences through FRET melting assays with our bioactive phenanthrolines. Concerning the stabilization of the parasitic G-quadruplex, it could be noticed that the best diphenylphenanthrolines **1** which exhibited an interesting stabilization profile were those substituted by the longest aminoalkylaminomethyl side chains. However, we found no correlation between the antiparasitic activity and selectivity of these derivatives and their binding to G-quadruplexes. These derivatives are therefore unlikely to be specifically cytotoxic via G-quadruplex binding. Moreover, it would be now interesting to enlarge the biological evaluation of these new diphenylphenanthrolines derivatives **1** by studying their mechanism of action through further investigations such as the inhibition of beta-hematin formation or of the apicoplast functions. Finally, the new substituted 2,9-bis[(substituted-aminomethyl)]-4,7-phenyl-1,10-phenanthrolines could open the way to new valuable medicinal chemistry scaffolding in the antiprotozoal domain.

## Figures and Tables

**Table 1:** Physical properties of ammonium oxalate salts **1a**–**p**.

Compound		Salt ^a^	mp (°C) ^b^	% Yield ^c^
**1a**	Orange crystals	4 (COOH)_2_	179–181	64
**1b**	Beige crystals	4 (COOH)_2_	161–163	60
**1c**	Orange crystals	4 (COOH)_2_	111–113	56
**1d**	Beige crystals	4 (COOH)_2_	119–121	57
**1e**	Orange crystals	6 (COOH)_2_	185–187	81
**1f**	Yellow-orange crystals	6 (COOH)_2_	205–207	58
**1g**	Beige crystals	6 (COOH)_2_	207–209	63
**1h**	Beige crystals	4 (COOH)_2_	194–196	73
**1i**	Yellow crystals	4 (COOH)_2_	174–176	61
**1j**	Beige crystals	4 (COOH)_2_	192–194	69
**1k**	Beige crystals	4 (COOH)_2_	141–143	62
**1l**	Beige crystals	4 (COOH)_2_	145–147	61
**1m**	Orange crystals	2 (COOH)_2_	165–167	63
**1n**	Orange crystals	2 (COOH)_2_	164–166	74
**1o**	Beige crystals	2 (COOH)_2_	147–149	66
**1p**	Yellow crystals	2 (COOH)_2_	99–101	83

^a^ The stoichiometry and composition of the salts were determined by elemental analyses and obtained values were within ±0.4% of the theoretical values. ^b^ Crystallization solvent: 2-PrOH–H_2_O. ^c^ The yields only included the conversions into the ammonium oxalates.

**Table 2:** In vitro sensitivity of *P. falciparum*, *L. donovani* and *T. brucei brucei* strains to compounds **1a**–**p** and cytotoxicity of these compounds in HepG2 cells.

Compound	*P. falciparum Strains* IC_50_ Values (μM) ^a^		*L. donovani* IC_50_ Values (μM) ^b^	*Trypanosoma brucei brucei* IC_50_ Values (μM) ^c^	Cytotoxicity to HepG2 Cells CC_50_ Values (μM) ^d^
	W2	3D7		*Trypanos Antat 1.9*	
CQ ^e^	0.40 ± 0.04	0.11 ± 0.01	n.d. ^h^	n.d. ^h^	30
MQ ^e^	0.016 ± 0.002	0.06 ± 0.003	n.d. ^h^	n.d. ^h^	n.d. ^h^
Pentamidine^f^	n.d. ^h^	n.d. ^h^	5.5 ± 0.80	0.0002 ± 0.00006	2.3 ± 0.50
Amphotericin B ^f^	n.d. ^h^	n.d. ^h^	0.1 ± 0.04	n.d. ^h^	8.8 ± 0.60
Suramine ^g^	n.d.^h^	n.d. ^h^	n.d. ^h^	0.03 ± 0.003	n.d. ^h^
Fexinidazole ^g^	n.d.^h^	n.d. ^h^	n.d. ^h^	0.59 ± 0.039	n.d. ^h^
Eflornithineg	n.d. ^h^	n.d. ^h^	n.d. ^h^	15.19 ± 0.64	n.d. ^h^
Doxorubicin	n.d. ^h^	n.d. ^h^	n.d. ^h^	n.d. ^h^	0.06 ± 0.02
**1a**	1.00 ± 0.34	1.80 ± 0.67	>12.5 ^i^	0.67 ± 0.03	6.52 ± 0.61
**1b**	6.13 ± 1.63	21.19 ± 7.72	>12.5 ^i^	0.57 ± 0.07	5.96 ± 0.20
**1c**	0.03 ± 0.003	10.31 ± 2.13	>12.5 ^i^	0.86 ± 0.16	10.43 ± 0.70
**1d**	3.51 ± 0.52	4.79 ± 0.50	>12.5 ^i^	1.42 ± 0.10	3.35 ± 0.33
**1e**	0.92 ± 0.18	2.16 ± 0.32	>12.5 ^i^	0.45 ± 0.03	10.54 ± 1.27
**1f**	11.27 ± 2.93	21.54 ± 4.10	>12.5 ^i^	0.97 ± 0.11	8.62 ± 0.50
**1g**	2.48 ± 0.49	8.19 ± 3.53	>12.5 ^i^	0.96 ± 0.13	16.75 ± 0.60
**1h**	0.17 ± 0.05	0.67 ± 0.21	4.50 ± 2.20	0.22 ± 0.06	5.89 ± 0.53
**1i**	0.04 ± 0.01	1.45 ± 0.58	>12.5 ^i^	0.29 ± 0.02	6.29 ± 1.06
**1j**	2.60 ± 0.55	1.59 ± 0.59	2.52 ± 0.20	0.20 ± 0.02	1.64 ± 0.14
**1k**	1.65 ± 0.36	3.18 ± 0.68	>12.5 ^i^	0.77 ± 0.10	4.29 ± 0.52
**1l**	0.03 ± 0.01	9.91 ± 1.73	>12.5 ^i^	1.13 ± 0.06	15.17 ± 1.56
**1m**	0.07 ± 0.01	0.47 ± 0.12	>12.5 ^i^	0.63 ± 0.01	4.34 ± 0.31
**1n**	2.54 ± 0.36	0.71 ± 0.11	2.67 ± 0.80	0.43 ± 0.03	3.10 ± 0.19
**1o**	1.87 ± 0.58	0.73 ± 0.19	2.69 ± 0.60	0.21 ± 0.01	19.11 ± 2.11
**1p**	0.145 ± 0.04	1.02 ± 0.35	>12.5 ^i^	0.65 ± 0.05	3.04 ± 0.19

^a^ Values were measured against CQ-resistant and mefloquine-sensitive strain W2 and the CQ-sensitive and MQ-resistant strain 3D7. ^b^ IC_50_ values were measured against the promastigotes of *Leishmania donovani* strain. The IC_50_ (µM) values correspond to the means +/− standard deviations from three independent experiments. ^c^ IC_50_ values were measured against the slender bloodstream trypomastigotes of *Trypanosoma brucei brucei* AnTat 1.9 strain. The IC_50_ (µM) values correspond to the means +/− standard deviations from three independent experiments with each concentration tested in duplicate in all experiments. ^d^ CC_50_ values were measured against HepG2 cells. The CC_50_ (µM) values correspond to the means +/− standard deviations from three independent experiments. ^e^ CQ and MQ were used as antiplasmodial compounds of reference. ^f^ Pentamidine and amphotericin B were used as antileishmanial compounds of reference. ^g^ Suramine, pentamidine, fexinidazole, and eflornithine were used as antitrypanosomal compounds of reference. ^h^ n.d.: not determined. ^i^ Molecule was not tested at higher concentrations (not active).

**Table 3:** Selectivity indexes of compounds **1a**–**p**.

Compound			Selectivity Index ^a^	
	HepG2/W2	HepG2/3D7	HepG2/*L. donovani*	HepG2/*Tryp.*
CQ	75	272	n.d. ^b^	n.d. ^b^
Pentamidine	n.d. ^b^	n.d. ^b^	n.d. ^b^	11,500
Amphotericin B	n.d. ^b^	n.d. ^b^	88.0	n.d. ^b^
**1a**	6.52	3.62	n.d. ^b^	9.73
**1b**	0.97	0.28	n.d. ^b^	10.46
**1c**	347.67	1.01	n.d. ^b^	12.13
**1d**	0.95	0.70	n.d. ^b^	2.36
**1e**	11.46	4.88	n.d. ^b^	23.42
**1f**	0.76	0.40	n.d. ^b^	8.89
**1g**	6.75	2.04	n.d. ^b^	17.45
**1h**	34.64	8.79	1.31	26.77
**1i**	157.25	4.34	n.d. ^b^	21.69
**1j**	0.63	1.03	0.65	8.20
**1k**	2.60	1.35	n.d. ^b^	5.57
**1l**	505.7	1.53	n.d. ^b^	13.42
**1m**	62.0	9.23	n.d. ^b^	6.89
**1n**	1.22	4.37	1.16	7.21
**1o**	10.22	26.18	7.10	91.0
**1p**	20.97	2.98	n.d. ^b^	4.68

^a^ SI was defined as the ratio between the CC_50_ value on the HepG2 cells and the IC_50_ value against the *P. falciparum* W2 or 3D7 or *Trypanosoma brucei brucei* strains. ^b^ n.d.: not determined.

**Table 4:** FRET melting values for compounds **1a**–**p (**2 μM) with FPf1T, FPf8T, FtryBT, F21T and FdxT (0.2 µM) in K^+^ conditions.

Compound	ΔT_m_ (°C) ^a^		ΔT_m_ (°C) ^a^		ΔT_m_ (°C) ^a^		ΔT_m_ (°C) ^a^		ΔT_m_ (°C) ^a^	
	FPf1T		FPf8T		FtrypBT		F21T		FdxT	
PhenDC3	24.6	±0.1	24.7	±0.2	19.2	±0.2	26.3	±0.1	0.1	±0.2
CQ	1.9	±0.1	2.4	±1.2	n.d. ^b^		2.4	±1.1	n.d. ^b^	
MQ	3.1	±0.5	6.6	±2.3	n.d. ^b^		2.6	±0.5	n.d. ^b^	
**1a**	1.8	±0.3	1.6	±0.2	1.9	±0.1	1.8	±0.4	−0.3	±0.2
**1b**	7.4	±0.6	7.9	±0.3	8.7	±0.6	12.8	±1.3	−0.3	±0.2
**1c**	8.9	±0.8	10.2	±1.0	10.6	±0.8	13.2	±0.4	−0.5	±0.4
**1d**	18.8	±0.2	20.5	±0.2	14.7	±0.7	18.1	±0.9	1.8	±0.2
**1e**	9.4	±1.0	10.5	±0.1	8.0	±0.4	9.6	±0.6	−1.6	±0.2
**1f**	8.8	±0.5	10.9	±0.9	9.1	±0.5	10.8	±0.6	−0.5	±0.1
**1g**	13.4	±0.7	14.5	±1.2	12.2	±1.7	11.2	±0.9	−0.5	±0.1
**1h**	1.8	±0.4	1.9	±0.2	2.2	±0.1	2.0	±0.2	−0.9	±0.4
**1i**	6.7	±0.5	6.7	±0.6	6.0	±0.9	8.2	±0.1	−1.5	±0.4
**1j**	5.6	±0.6	7.3	±2.4	7.2	±0.6	7.7	±2.1	0.2	±0.1
**1k**	12.8	±0.7	14.0	±0.3	8.3	±1.0	12.0	±0.7	0.4	±0.3
**1l**	15.4	±0.9	14.2	±0.9	11.6	±0.9	14.5	±0.4	0.7	±0.2
**1m**	1.3	±0.2	1.0	±0.2	1.8	±0.6	1.0	±0.5	−0.2	±0.1
**1n**	2.9	±0.4	5.3	±0.4	5.2	±0.3	4.6	±0.6	−0.3	±0.1
**1o**	2.9	±0.1	5.2	±0.1	5.3	±0.7	3.9	±0.3	−0.3	±0.2
**1p**	3.5	±0.3	3.9	±0.7	3.7	±0.3	3.6	±0.3	−1.1	±0.2

^a^ ΔT_m_ of FPf1T, FPf8T, FtryBT, F21T and FdxT (0.2 μM) were recorded in 10 mM lithium cacodylate (pH 7.2), 10 mM KCl, 90mM LiCl. PhenDC3 was tested at 0.5 μM, whereas CQ and MQ at 1 μM. Error margins correspond to SD of three replicates. ^b^ n.d.: not determined.

**Table 5:** Dissociation constants (µM) obtained by native mass spectrometry experiments.

Ligands		*K*_d_ (µM) ^a^				
Name	Stoichiometry	Pf1	Pf8	24TTG	24nonG4	DK66
**1c**	1	23	3	44	9	110
**1d**	1	8	3	38	11	53
**1l**	1	10	2	34	8	55
**1m**	1	n.b.	83	n.b.	n.b.	n.b.
**1c**	2	n.b.	50	28	<LOQ	n.b.
**1d**	2	24	16	62	<LOQ	n.b.
**1l**	2	37	59	18	<LOQ	n.b.

^a^ n.b.: no binding detected, <LoQ: binding detected but below limit of quantitation.

## Data Availability

Not applicable.

## Supplementary Materials

The following supporting information can be downloaded at: https://www.mdpi.com/article/10.3390/pathogens11111339/s1, Figure S1: Circular dichroism spectra of Pf1, Pf8, 24TTG and 24nonG4 in absence (gray) or presence of one (blue) or two (red) molar equivalents of molecules **1c**, **1d** and **1l**; Figure S2: Folded fraction as a function of the temperature for the oligonucleotides 24TTG, Pf1 and Pf8 (cooling ramp: blue, heating ramp: red), obtained by UV-melting following the protocol previously described [55]; Figure S3: Native ESI-MS spectra showing the 5- charge state species (M: monomer, D: dimer). Free DNA (gray), 1:1 complexes (green) and 2:1 complexes (orange) are annotated on the spectra. Potassium cation stoichiometries are indicated with **o** (0), • (1K^+^) and •• (2K^+^).
