# Supplementary material for: Design, Synthesis, and Antiprotozoal Evaluation of New Promising 2,9-Bis[(substituted-aminomethyl)]-4,7-phenyl-1,10-phenanthroline Derivatives, a Potential Alternative Scaffold to Drug Efflux
Source: Pathogens. 2022 Nov 13;11(11):1339. doi: 10.3390/pathogens11111339 (PMC9699089; doi:10.3390/pathogens11111339)
Supplement: Supplementary file 1 [file pathogens-11-01339-s001.zip › pathogens-1982076-supplementary.pdf]

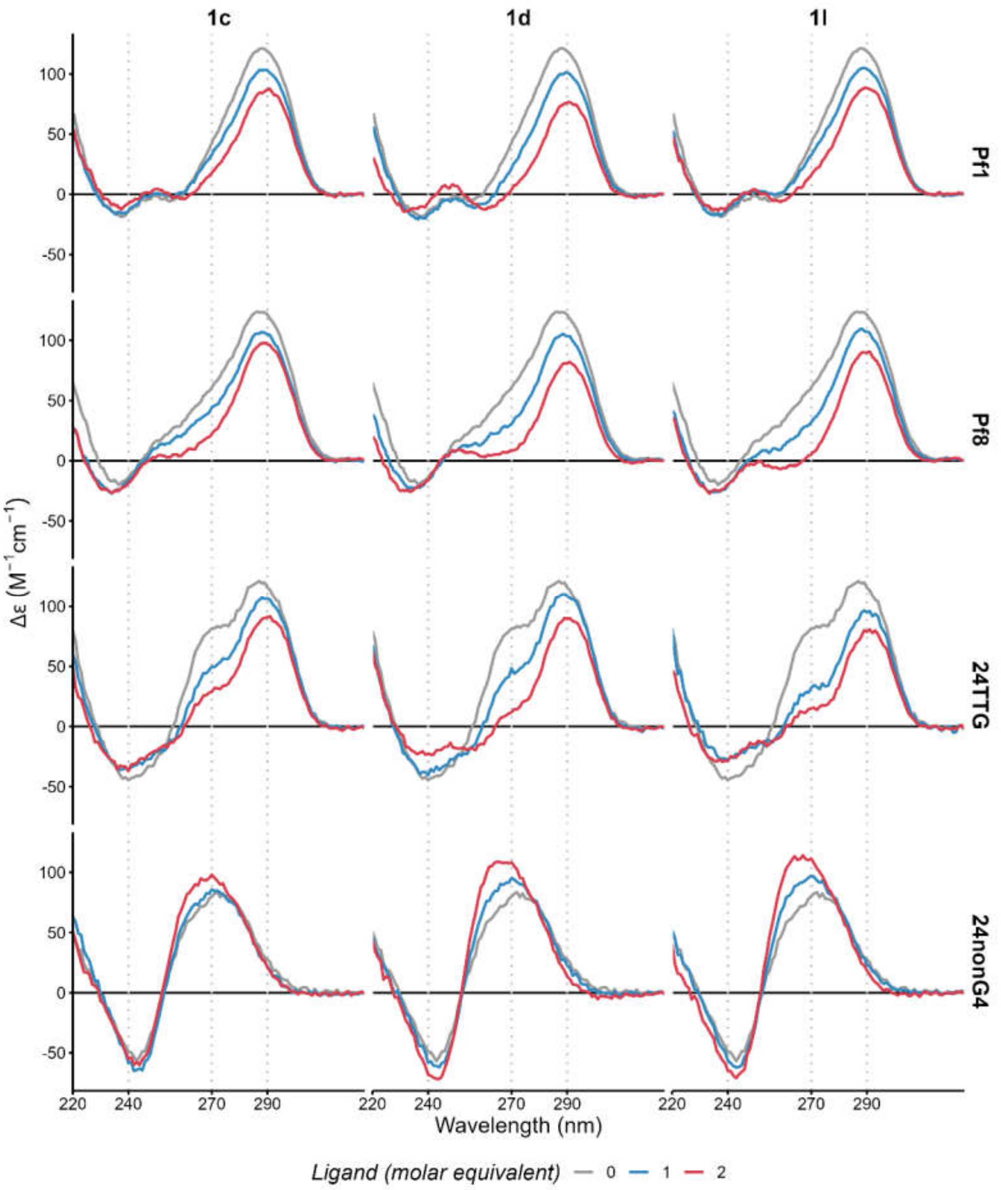

Figure S1. Circular dichroism spectra of Pf1, Pf8, 24TTG and 24nonG4 in absence (grey) or presence of one (blue) or two (red) molar equivalents of molecules **1c**, **1d** and **1l**.

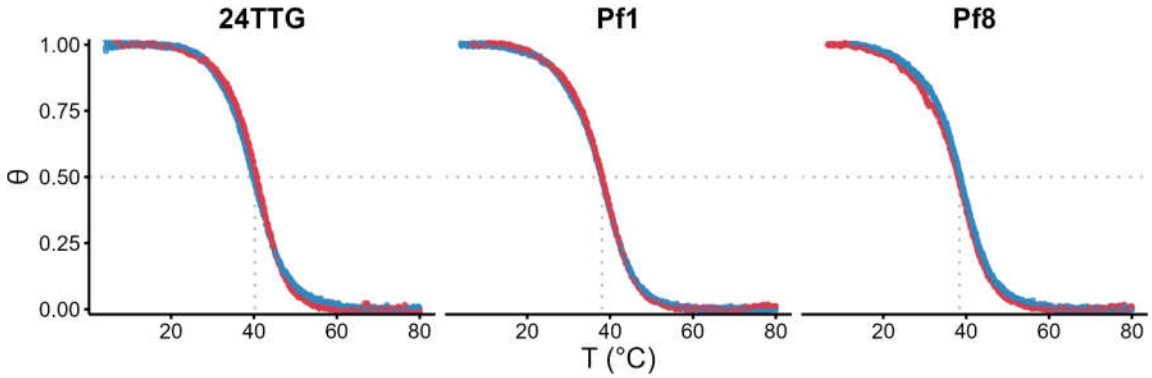

Figure S2. Folded fraction as a function of the temperature for the oligonucleotides 24TTG, Pf1 and Pf8 (cooling ramp: blue, heating ramp: red), obtained by UV-melting following the protocol previously described [50].

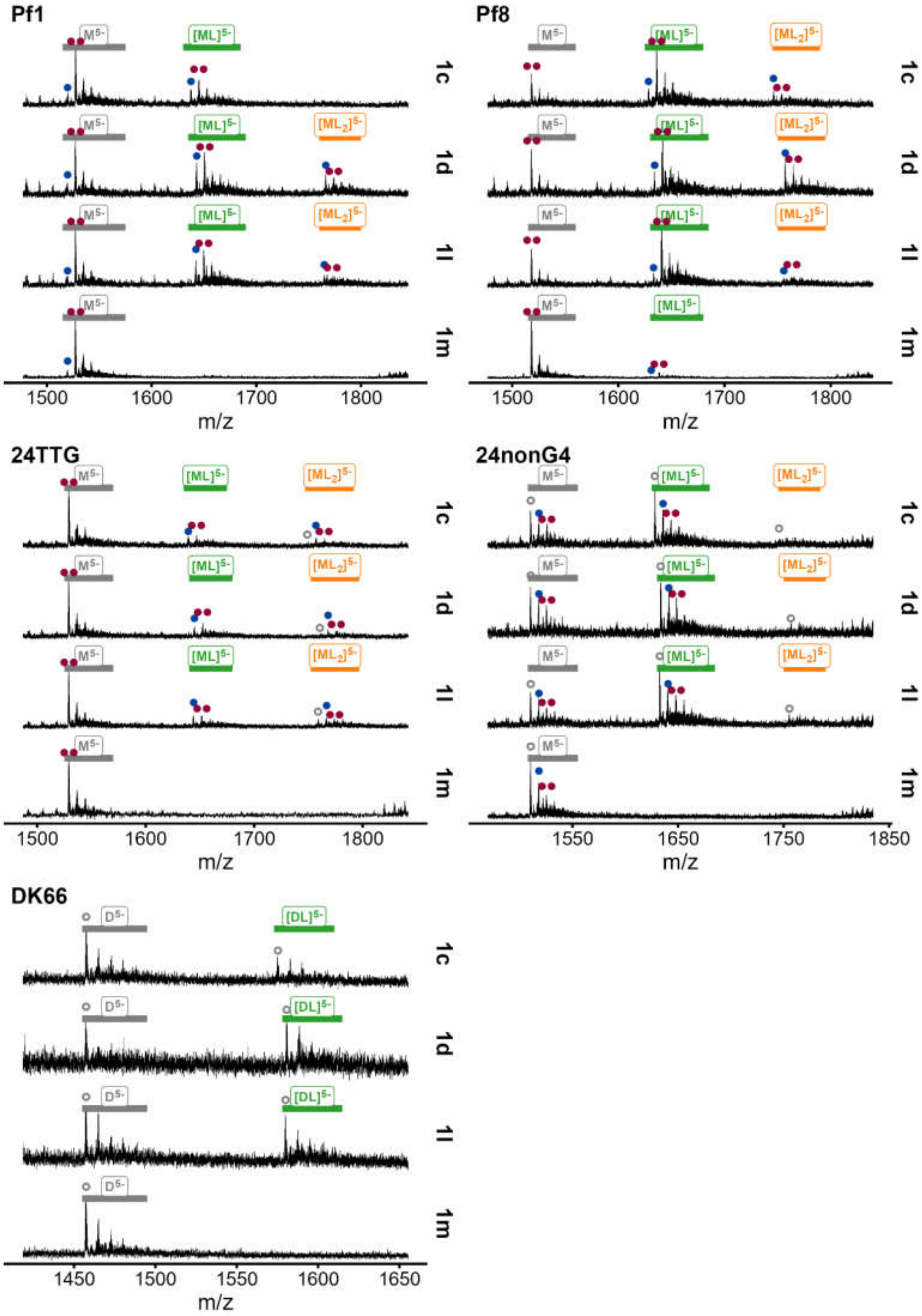

Figure S3. Native ESI-MS spectra showing the 5- charge state species (M: monomer, D: dimer). Free DNA (grey), 1:1 complexes (green) and 2:1 complexes (orange) are annotated on the spectra. Potassium cation stoichiometries are indicated with **o** (0), • ($1K^+$) and •• ($2K^+$).
